# 6-Month-Old Infants’ Sensitivity to Contingency in a Variant of the Mobile Paradigm With Proximal Stimulation Studied at Fine Temporal Resolution in the Laboratory

**DOI:** 10.3389/fpsyg.2021.610002

**Published:** 2021-03-05

**Authors:** Sergiu Tcaci Popescu, Alice Dauphin, Judith Vergne, J. Kevin O’Regan

**Affiliations:** Integrative Neuroscience and Cognition Center, CNRS, Universite de Paris, Paris, France

**Keywords:** mobile paradigm, sensorimotor contingency detection, limb differentiation, non-conjugate reinforcement, conjugate reinforcement, operant conditioning, sense of agency, causal learning

## Abstract

Infants’ ability to monitor “sensorimotor contingencies,” i.e., the sensory effects of their own actions, is an important mechanism underlying learning. One method that has been used to investigate this is the “mobile paradigm,” in which a mobile above an infant’s crib is activated by motion of one of the infant’s limbs. Although successfully used in numerous experiments performed in infants’ homes to investigate memory and other types of learning, the paradigm seems less robust for demonstrating sensitivity to sensorimotor contingencies when used in the laboratory. One purpose of the present work was to show that certain changes to the mobile paradigm would make it easier for infants to show their sensitivity to the contingency in the lab. In particular, we used proximal stimulation on infants’ wrists instead of the usual mobile, and our stimulation was coincident with the limbs that caused it. Our stimulation was either on or off, i.e., not modulated by the amount the infant moved. Finally, we used a “shaping” procedure to help the infant discover the contingency. In addition to these changes in the paradigm, by analyzing infants’ limb activity at 10-s resolution instead of the usual 1-min resolution, we were able to show that infants’ sensitivity to the contingency became apparent already within the first minute of establishment of the contingency. Finally, we showed how two alternate measures of sensitivity to contingency based on probability of repeated movements and on “stop and go” motion strategies may be of interest for future work.

## Introduction

Numerous authors from different fields of psychology have stressed that an infant’s sensitivity to “sensorimotor contingencies,” i.e., to the sensory results of the infant’s own actions, is a major mechanism underlying infant development (Piaget, 1936; Watson, 1966; Edelman, 1987; Gibson, 1988; Thelen, 1995), be it in the development of motor learning, exploration, awareness of the body, sense of agency, social behavior and language.

The present, methodological, study is concerned with a particular paradigm that has been widely used in this literature since its first use by Watson and Ramey (1969, 1972) and Rovee and Rovee (1969), namely the “mobile paradigm.”

In the basic version of this paradigm, a ribbon attached to an infant’s limb is connected to a mobile placed above the infant’s crib, so that when the infant moves the limb, this causes corresponding (“conjugate”) motion of the mobile (Rovee and Rovee, 1969). In variants of the paradigm, other, possibly non-conjugate types of stimulation (e.g., appearance of colored lights in Millar, 1972), can be triggered by limb motion, and the particular limb motion required may be more or less precisely defined (e.g., knee flexion at 85° in Angulo-Kinzler et al., 2002), or other modes of triggering can be used (e.g., head motion in Watson and Ramey, 1969, 1972). The conditions used as controls in the paradigm can involve a group of subjects that receives the same stimulation but without this being contingent on their movement, or a condition where movement of the reinforced limb is compared to a similar non-reinforced limb, or where reinforcement involves different task demands. As a measure of an infant’s sensitivity to the contingency researchers generally use the amount of stimulation that the infant provokes compared to a non-contingent control, but other measures are also used, for example observation of specific reinforced movement patterns compared to a control.

Early work by Watson and Rovee-Collier had shown the effectiveness of the mobile paradigm in demonstrating young infants’ sensitivity to sensorimotor contingencies. Later work then predominantly used the paradigm as a tool in order to test infants’ learning, memory, recognition, or categorization abilities, leading to a very considerable literature (for comprehensive overviews see Rovee-Collier and Gekoski, 1979; Jacquey et al., 2020a). Multiple publications have shown, for example, the importance of an infant’s age in determining sensitivity to contingency, the role of environmental context, of previous experience, of the specificity of the movement that provokes the stimulation, the effect of delays between movement and stimulation, the effect of changes in the stimulus, etc.

As noted recently by Merz et al. (2017), an interesting methodological aspect of these studies is that the majority were conducted at infants’ homes, rather than in a laboratory setting. The reason for this may be the difficulty of getting robust effects in the laboratory. Indeed in one laboratory study, Rovee-Collier et al. (1978) had remarked “A disturbing feature of the experiment was the high rate of subject attrition (70% by the final phase) relative to that typically encountered when infants are tested with similar procedures in their own homes (0–5%) […]. In general, infants tested in the laboratory appeared to be less responsive to the mobile along a variety of dimensions (response vigor, initial attention, etc.) than home-tested infants […]” (p. 327).

Consideration of those studies performed in the laboratory where limb motion determines stimulus activation confirms first that there are few such studies, and second that some of these have rejected a large number of infants, with attrition rates of up to 74%. In particular, Watanabe, Taga and their co-workers conducted an extensive series of experiments on 2- to 4-month-old infants in the lab where many infants did not complete the experiment because they cried, sucked their fingers, rolled over, or became drowsy (Watanabe and Taga, 2006: 48 retained, 100 rejected; Watanabe et al., 2011, Experiment 1: 64 retained, 76 rejected; Watanabe and Taga, 2011: 186 retained, 106 rejected; Kato et al., 2013: 77 retained, 12 rejected; Watanabe and Taga, 2009: 54 retained, 153 rejected). Angulo-Kinzler et al. (2002) is another study showing the difficulty of finding infants that can learn a contingency, with 11 out of 29 3-month-old infants being unable to “learn the contingency” in the sense of attaining the criterion of 1.5 times baseline activity usually used in the literature (but note that amount of activity is not the only way sensitivity to contingency might manifest itself, and these authors did show effects on infants’ specific motor patterns).

Some other studies conducted in the laboratory show attrition rates that were somewhat better (ranging from 0 to 19%), but where sensitivity to contingency was restricted to older infants, was not strong, or remained debatable. Thus, Millar (1975) was not able to demonstrate sensitivity to contingency at 5 months, only at 7 months. Lewis et al. (1985) noted that their 2-, 4- and 6-month-old infants increased their response rate slightly more over the experiment than did infants from the non-contingent group. However, the authors did not evaluate the statistical significance of these effects [although Sullivan and Lewis (1989) using a similar procedure reported significant effects]. Merz et al. (2017) found that 71% of their 4-month-old infants increased their kicking rate to more than 1.5 times the baseline. But without comparison to a non-contingent control group this apparent sensitivity to the contingency might have been partially caused by increased arousal over the course of the experiment.

As seen from this overview of lab-based studies we could find in the literature, and confirming Rovee-Collier et al.’s (1978) remark, it seems that demonstrating infants’ sensitivity to a contingency with the mobile paradigm or its variants is more problematic in the laboratory than when the experiment is done at home. Attrition rate is sometimes notably higher, the presence of sensitivity is sometimes debatable and only evident in the oldest age groups or only present as a trend.

Yet, as pointed out by Merz et al. (2017), being able to use the mobile paradigm in the laboratory would open the door to experimentation with a broader range of participants and conditions. In our team, with a view to applications in robotics, we have been interested in studying more closely how infants’ sensitivity to contingencies generalizes to variations in the response demanded, in the stimuli, or in the stimulation modality. To obtain such flexibility in the experimental conditions we have been experimenting with a variant of the mobile paradigm where limb movements are measured in real time by wireless-enabled accelerometers, and where computer-controlled visual or auditory stimulation can be easily varied (Jacquey et al., 2020b). However, like the studies reviewed above, we also had difficulties showing sensitivity to contingencies. Here we recall our results from that study, since the experiment described in the present article will be referencing that study.

In Jacquey et al. (2020b) we studied 104 infants aged 4, 6, and 8 months using a contingency where movement of one arm triggered proportional (i.e., “conjugate”) motion of a cartoon face on a screen in front of the infant. We had a fairly low attrition rate (104 infants included, 8 infants rejected for fussiness), perhaps because our experiment lasted only 4 min and we did not exclude infants on the basis of a learning criterion as is usually done. We found evidence for sensitivity to the contingency, but it was weak. In particular, we found that global arm activity in the contingent condition did not differ significantly from activity in a non-contingent control condition where the stimulation occurred with matched frequency but not under the infant’s control. We did, however, observe a significant effect of contingency on the *rate of increase* of global arm activity over the experiment: infants in the contingent condition increased their activity more rapidly than infants in the non-contingent condition. Further evidence for sensitivity to the contingency derived from comparison of the activity of the arm whose motion produced the stimulation (the “connected” arm) to activity of the arm whose motion did not have any effect (the “unconnected” arm). We found that there was a significant main effect, with the “connected” arm moving more than the “unconnected” arm (independently of whether it was the right or left arm). This finding is consistent with Watanabe, Taga and collaborators’ work (e.g., Watanabe and Taga, 2009) showing that infants can sometimes differentiate their limbs and favor movement of the “connected” limb. However, we did not find the effect of age that these authors found: arm differentiation did not improve with age. In fact, even the difference in rate of increase in global activity between contingent and non-contingent conditions did not show an influence of age. If anything, the 6-month-old infants seemed more sensitive to the contingency than the other age groups.

Given these difficulties in Jacquey et al. (2020b), and given the questions raised by our survey of other authors’ lab-based experiments with the mobile paradigm and its variants, the purpose of the present, methodological, work was to attempt to find ways to increase the robustness of our variant of the mobile paradigm so that sensitivity to contingency could be more reliably demonstrated in the lab. One strategy we investigated was to modify the conditions used so as to increase the infant’s ability to detect and exploit the contingency. In Section “Modification of the Mobile Paradigm” we argue for the changes we chose to make. A second strategy was to reconsider the way sensitivity to contingency is measured. In Section “Additional Measures of Sensitivity to Contingency” we present the idea of using higher temporal resolution, and of using statistical measures derived from the work of Watson (1979, 1984, 1985) and Movellan (2005) and Butko and Movellan (2010).

### Modification of the Mobile Paradigm

A series of unpublished pilot experiments by Jacquey (2019) had suggested that making the following changes in the procedure would increase infants’ sensitivity to the contingency.

One change we made was that instead of using a “conjugate” stimulus whose movements were proportional to the amount of limb movement produced by the infant, we used a binary stimulation that could either be on or off, and a high threshold for the motor action required to trigger it. The choice of a binary stimulus is supported by Zwicker et al. (2012) who found that adults are better at detecting a time delay between a movement of their unseen hand and a delayed recording of the movement when the movement is discrete—with a sudden onset and offset—as opposed to being continuous. A similar advantage of suddenness was also observed by Watson (1979), who found that infants were more sensitive to a contingency and learned faster in a condition requiring a strong foot kick than a light foot kick. Anecdotally, the popularity of peek-a-boo and rattle toys support these experimental choices. Our choice of using a high motor action threshold to trigger the stimulation was motivated by the idea that the infant should be unlikely to produce the action accidentally. To facilitate the discovery of the contingency, our experiment used a “shaping” regime that started with a low threshold and that was increased in a few steps to reach the high value used throughout the rest of the experiment.

Another change we made here compared to Jacquey et al. (2020b) was the fact that we presented the stimuli in a region of space that was within infants’ reach, that is, in their proximal space as opposed to distal space as done in the mobile paradigm. This choice is supported by studies showing that infants aged 5–6 months actively explore only the objects that are within reach (as evidenced by posture preparation and reaching) and that they become disinterested or even resistant to exploring outside this region (Rochat and Goubet, 1995; Rochat et al., 1999). Our stimuli were also spatially lateralized—instead of being presented on a centrally located screen, they were presented on devices attached to the infants’ wrists. The fact that each stimulus was lateralized and graspable was expected to facilitate detection of the contingency, since grasping allows multiple additional affordances that infants could explore actively at this age.

Another important aspect of our stimulation, different from Jacquey et al. (2020b), was the fact that the stimulus was presented on the limb that the infant was moving. This spatial coincidence between action and resulting effect is a common property of many everyday body-linked contingencies, and so we thought it would favor detection of the contingency. Also the spatial coincidence allows the infant’s attention to simultaneously capture its limb movements and the accompanying feedback (Millar and Schaffer, 1972). An additional point worth mentioning is that our device was somewhat heavier (about 45 g) than the bracelet used in our previous study (about 20 g), and so provided more tactile and proprioceptive feedback when the infants moved their arms. This might be analogous to a small amount of tactile feedback that infants in the classic mobile paradigm would have felt from the ribbon that was tied to their limbs, and that was connected to the mobile.

### Additional Measures of Sensitivity to Contingency

A second strategy we adopted to try to show more robust sensitivity to the contingency in our experiment was to rethink how sensitivity to contingency is usually measured.

In most studies using the mobile paradigm, researchers base their analyses on the evolution of motor activity over successive blocks, each of duration ranging from half a minute (e.g., the studies by Watanabe, Taga and their collaborators cited above) to as much as 3 min (Millar, 1975). Yet in some studies sensitivity to contingency manifests itself within 30 s of a contingency being established (Watanabe and Taga, 2011). If such effects appear and rapidly extinguish, they would be missed using analyses having long temporal averaging. Therefore, in the study to be presented here, in addition to the usual 1-min bins used for pooling motor activity, we analyzed our data at a finer temporal resolution (10-s bins and in some cases 1-s bins).

In addition to using finer temporal resolution, we investigated two further ways to show sensitivity to contingency which, instead of measuring limb activity over the experiment, make use of probability measures. One way is based on work by Watson (1979, 1984, 1985), who had proposed that if an infant detects an action-stimulus contingency, the infant might explore and re-test for the presence of this contingency by making further movements. As a result, the probability of subsequent action-stimulus events during a specific time interval—during which the infant retains information about the action-stimulus contingency—should be higher than during other times, and also compared to the case when an infant did not detect the relation between its motor action and the sensory outcome.

Another way to show sensitivity to contingency was proposed by Butko and Movellan (2010) and Movellan (2005) who developed Watson’s idea and applied it to contingency detection in the context of social communication with a simple artificial agent. Their results showed that 10-month-old infants, before language acquisition, actively probe the reactions of the agent by alternating between stopping and starting their vocalizations at an optimal rhythm for contingency detection. Analogously, in our paradigm, infants who suspect the existence of a contingency might be expected to test their hypothesis by adopting alternate moving and freezing behavior. Such stop-and-go behavior would be an efficient strategy to obtain the reward because this behavior maximizes stimulation while minimizing motor effort. Using such an efficient motor strategy—that does not lead to a global increase of activity but which nevertheless increases the frequency of rewards—has been observed by Angulo-Kinzler et al. (2002). If infants do use such a stop-and-go behavior to test for contingency, the behavior should be visible in the statistical distribution of movement quantity. It would appear as excess activity, compared to non-contingent controls, at the low and high ends of the distribution: more very small movements or “stops” at the low end, and more large movements or “goes” at the high end. Furthermore, if the infants differentiate between their arms, this increase in extreme values in the distribution of activity would be expected to occur more for the contingent arm that causes the stimulation than for the non-contingent arm.

### Summary of Purpose of the Experiment and Hypotheses

To summarize, the present work first investigates whether certain changes to our variant of the mobile paradigm will provide clearer evidence for sensitivity to contingencies than what we observed in Jacquey et al. (2020b). We chose to use a contingency that involved a non-conjugate (i.e., either on or off) stimulus triggered by a sharp action that was unlikely to be due to chance, presented in proximal space, and spatially coincident with the action that produced it. To demonstrate the efficiency of our modified paradigm, we chose to test 6-month-old infants, and to compare the results to the 6-month-old age group in Jacquey et al. (2020b), since this group had shown the clearest effects. We kept the mode of recruitment, the environmental conditions (place, booth, and lighting), the use of bracelets on wrists, and the number of infants very similar if not common between the two experiments, so that the results of our comparison could reasonably be attributed to our changes in the experimental paradigm. We also used the same conditions as in that experiment: an experimental group where the stimulation was presented contingent on motions of one or other of the infant’s arms; and a non-contingent control group where matched stimulus activity was presented to infants without being contingent on their arm movements. Also as before, we measured both arms’ activity, and compared activity of “connected” and “unconnected” arms to assess whether infants were able to differentiate their arms by favoring activation of the arm that controlled the stimulation. Showing evidence of such differentiation is another, within-Subject, way to demonstrate that infants are sensitive to the contingency, in addition to the between-Subject comparison of contingent to non-contingent groups. We expected that if our paradigm modifications improved sensorimotor contingency detection as compared to Jacquey et al.’s (2020b) group of 6-month-old infants, we would observe clearer evidence for sensitivity to contingencies both when the contingent condition is compared to the non-contingent condition, and when activity of the “connected” arm is compared to activity of the “unconnected” arm. In particular, we expected that the proportion of variance accounted for in the data by the contingency/non-contingency and by the connected/unconnected factors would be higher than in Jacquey et al. (2020b), and that effects of contingency would emerge earlier in the time course of our experiment than in that study.

The second aim of our study was to pilot some possibly more sensitive measures of contingency detection. One method involved analyzing our own data and re-analyzing the data of Jacquey et al. (2020b) at finer temporal resolution. Another method involved evaluating Watson’s (1979, 1984, 1985) idea that infants might systematically repeat their actions when they suspect that their actions create a stimulation. Related to this, following work by Movellan (2005) and Butko and Movellan (2010), another measure we explored involved testing whether infants adopt a “stop-and-go” strategy to explore the contingency. We did this by considering if there were more extreme values in the distributions of limb activity when there was a contingency than when not.

## Materials and Methods

### Apparatus and Stimuli

Two bracelets were constructed using BBC micro:bit microcomputers^1^ that were fitted with MI-power boards (5610)^2^ to enable wireless mode. Each bracelet included an accelerometer, a buzzer and display. Acceleration was measured along three orthogonal axes at about 9 Hz. The built-in display consisted of a 17 mm × 17 mm grid of 5 × 5 individually programmable red LEDs with 10 possible intensity levels. The two bracelets communicated wirelessly. Each bracelet weighed about 45 g.

Stimulation was presented to the infants via the bracelets, one worn on each wrist. To ensure salient stimuli, we combined audio and visual sensory feedback; also, to maintain the infants’ interest, we used one of 6 different possible dynamically changing light and sound patterns, chosen randomly at each triggering event, and displayed simultaneously on both bracelets. The sounds emitted by the bracelet could consist of a combination of three to five tones selected among three different frequencies (263, 330, and 392 Hz) with the same intensity (∼60 dB at a distance of ∼20 cm). The duration of each pattern was about 0.5 s. The same stimulation was used on both bracelets so as to ensure that differences between movements of the two arms could not be attributed to differences in audiovisual stimulation between the two arms, and could only be the result of the existence of a contingency.

**FIGURE 1:**
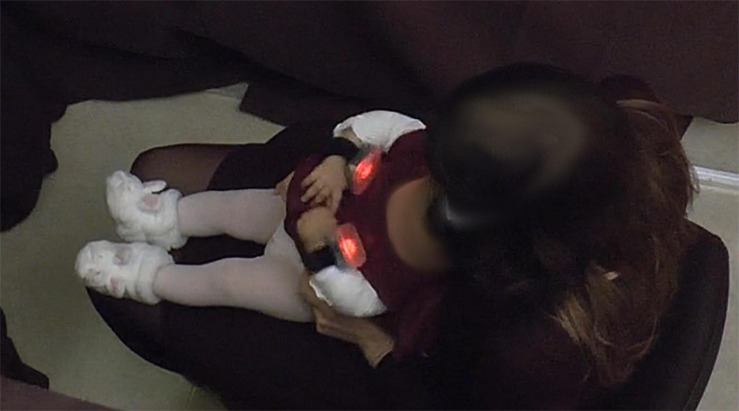
An extract from the video capture of an experimental session, viewed from above. The picture shows one moment in the 0.5-s dynamic visual stimulation emitted by both bracelets. The two bracelets emitted a dynamic auditory pattern at the same time. The face of the infant and the head of the adult have been blurred.

Figure 1 shows an infant wearing the bracelets with the stimulation turned on.

### Design

There were two experimental conditions. In the *Contingent* condition, both bracelets briefly turned on when the acceleration of one of the infant’s arms—the *Connected* arm—exceeded a threshold; the movements of the other arm—the *Unconnected* arm—did not trigger the stimulus. Initially set at 1.28 g m/s^2^ (g is the acceleration due to gravity), the threshold was increased by 0.08 g m/s^2^ after each stimulation, and then after 4 steps was maintained at 1.6 g m/s^2^ for the rest of the experiment. This final value of the threshold was attained on average after 31 s into the experiment (*SD* = 21 s; median = 22 s; range 9–71 s; third quartile = 50 s). The mean delay between the acceleration of the Connected arm reaching the stimulus-triggering threshold and the actual start of stimulus on the Connected arm was 37 ms (*SD* = 17 ms); on average the stimulus on the Unconnected arm started 2 ms (*SD* = 4 ms) later relative to the Connected arm, with a maximum difference of 13 ms (these data are based on a per-frame video analysis of 60 stimuli shot at slow motion 240 frames per second). While a stimulus was on, the measured acceleration during that time was not used to trigger a subsequent stimulus. A subsequent stimulus could be triggered immediately after a previous one ended.

The *Non-Contingent* condition was run on a second group of infants after the first group completed the Contingent condition. In that way we were able to measure the frequency at which bracelets turned on in the Contingent condition, and create a Non-contingent control that had the same overall frequency of triggering the stimulation. Since the distribution of the frequencies of occurrence observed in the Contingent condition was bimodal, in the Non-contingent condition we sampled occurrences of triggering from binomial distributions with two similar success rates: about 0.11 and 0.26.

To test the question of sensitivity to contingency we used a between-subject comparison. We planned for half of the infants to see the Contingent condition, and half to see the Non-contingent condition. Additionally, to control for possible effects of handedness when addressing the question of arm differentiation, in the Contingent condition, the arm (left or right) where we attached the connected bracelet was counterbalanced across the subjects. We attributed the left and right arms alternately to the infants as they were recruited.

Our main interest was to test for the effect of the contingency between the movement of one arm and the resulting audiovisual stimulation. We took a gravity-corrected acceleration measure (see section “Acceleration Data Processing”) for each arm as a measure of arm activity and as the dependent variable.

### Procedure

Before the experiment, in a room adjacent to the experimental booth, the experimenter presented the turned-off bracelets to the infant for familiarization. The two bracelets were then attached to the infant’s wrists. They were attached quite firmly in order to minimize motion relative to the infant’s arm. The caregiver was shown how to hold the infant in order to allow arm movements; the caregiver was also told to look in front and not at the infant during the entire experiment and not to communicate with the infant unless the infant turned their face toward the caregiver’s face, in which case the caregiver was told to briefly reassure the infant (by a smile, gesture or vocalization).

After the instructions were given, infant and caregiver were invited into the dimly lit experimental booth, where a small experimental cabin (about 2 m × 1 m, height about 2 m) made of black cloth was located. The caregiver was seated on a chair in the middle of the booth holding the infant on their lap, as shown in Figure 1. Two video cameras recorded the experiment, one in front of the infant (at 15 frames/s) and one above the infant (at 50 frames/s).

At the start of the experiment, the bracelets produced a 0.5-s dynamical sequence of flashes and tones in order to capture the infant’s attention (audiovisual stimulus). During the experiment, and depending on the condition, either movements of one of the infant’s arms could turn on the bracelets and produce the programmed patterns of audiovisual stimulation, or such an activity pattern was triggered randomly and independently of the infant’s arm motion.

The entire experimental session, from the first attention-getting audiovisual stimulus to the end of recording, lasted about 5.5 min.

### Participants

Sixty infants were recruited from a database of interested local middle to upper-middle class families. Before participating in the experiment, caregivers gave their written informed consent. The experimental protocol was approved by the University Paris Descartes ethics committee.

The data for 23 infants had to be rejected: in the Contingent test condition, 10 infants did not complete the entire experiment (for at least 5 continuous minutes) because they cried (5) or became fussy for at least 30 s (5), and 5 were excluded because of problems with data recording; in the Non-contingent control condition, 4 infants did not complete the entire experiment because they cried (3) or became fussy (1), data for 2 infants were incomplete due to technical error, 1 infant was excluded as preterm, and 1 infant was excluded because the caregiver did not follow instructions and restrained the infant’s arms.

Data analysis was thus based on 37 infants aged 6 months, 20 in the Contingent condition (9 females and 11 males, mean age = 184.5 days, *SD* = 7.5 days, range = 172–200 days) and 17 in the Non-contingent condition (8 females and 9 males, mean age = 181.9 days, *SD* = 7.2 days, range = 174–201 days).

### Acceleration Data Processing

Each accelerometer provided the instantaneous acceleration along the *X*-, *Y-*, and *Z*-axes, including Earth’s gravity. Since the accelerometers could rotate with the arm, the gravity axis was not constant and we needed to preprocess the data in order to discount the influence of gravity. We assumed that generally the amplitude and direction of the gravity vector would not change appreciably over short time periods, and so could be canceled out by calculating (*x*,*y*,*z*)_*t* + 1_ − (*x*,*y*,*z*)_*t*_—the difference of the acceleration vectors at two successive sampling times (at sampling rate of ∼9 Hz, i.e., ∼111 ms). We then took the Euclidean norm of this vector difference (root mean square of the coordinates) as a measure of arm activity. In our re-analysis of data in Jacquey et al. (2020b), because sampling frequency was 5 times faster than in the present experiment, the vector difference was calculated between vectors spaced by five sampling periods, (*x*,*y*,*z*)_*t* + 5_ − (*x*,*y*,*z*)_*t*_—again over an interval of ∼111 ms.

In the cases where we wanted to have an estimate of both arms’ activity—what we call *pooled activity*—we pooled the activity for both arms and took statistical measures using the pooled distributions.

### Data Correction

Despite the initial synchronization of the clocks of the two bracelets, comparison with the video recordings revealed that there was sometimes a slight time drift in the timestamps of the data series of one bracelet relative to that of the other. To correct this divergence, we divided each data series into three parts and determined, for each part, the optimal time shift of one series relative to the other that maximized the correlation between the activation statuses (on or off) of the two data series. The mean time shift was ∼270 ms (*SD* ∼196 ms), the maximum absolute time shift was 812 ms. Note, however, that such shifts affected almost exclusively the timestamps of the recorded data (which could be resynchronized in the way we have described) and not the triggering of the stimulation nor the actual synchrony of the bracelets during the experiment. We verified this by checking the visual and audio synchrony (frame-by-frame analysis of videos, at 15 frames-per-second) for the 4 infants with the highest average difference between the times of data series of each arm (two infants from each condition) and found several cases of visual asynchrony, each lasting ∼17 frames corresponding to ∼1 s. These cases were observed in less than 4% of stimuli. We found no noticeable audio asynchrony at all. Another technical problem consisted in inaccurate communication between Connected and Unconnected bracelets, resulting in a small number of activations of only one of the two bracelets (in the Contingent condition: *M* = 3%, *SD* = 2%, maximum = 9%; in the Non-contingent condition: *M* = 1%, *SD* = 1%, maximum = 3%). These were corrected in the data analysis by considering activations shorter than 100 ms as noise, and by splitting activations longer than twice the duration of an activation (500 ms).

### General Statistical Processing of Data

In this section we will define some statistical aspects that are common to the analyses in the “Results” section.

Outlier values are defined using the first and third quartiles (Q1 and Q3) and the interquartile range (IQR = Q3 − Q1). *Far outliers* are values lying outside the interval of [Q1 − 3 IQR, Q3 + 3 IQR]. The average activity of one infant in the Non-contingent condition was far-outlying and was excluded from the analyses unless specified otherwise. In our re-analyses of the data from Jacquey et al. (2020b) we excluded the data of one 6-month-old infant in the Non-contingent condition with far-outlying values (also, in the additional analysis reported in Supplementary Material Section “10-s Resolution Reveals Effect of Age in Jacquey et al. (2020b)” the data of one 4-month-old infant in the Contingent condition was excluded for the same reason).

All reported *p*-values are rounded to the nearest thousandth. The statistical tests are considered significant at *p* < 0.05. Unless otherwise specified, the statistical tests for all reported comparisons are two-sided. Bonferroni correction was implemented for multiple comparisons. Regarding Student’s *t*-tests, when two samples had unequal variances, Satterthwaite approximation to the effective degrees of freedom was applied.

In the reported analyses of variance with repeated-measure factors, when the result of Mauchly’s tests shows that the assumption of sphericity was not met, the degrees of freedom were corrected using Greenhouse–Geisser correction, this is why their values include decimal-place digits.

The analyses of variance (ANOVA) and generalized eta-squared reported here were done in R (R Core Team, 2020), using the functions *ezANOVA* from package *ez* (Lawrence, 2016). In doing our analysis of variance on data with unbalanced design, we followed the recommendations by Keppel and Wickens (2004): using the type III sum of squares.

Generalized eta-squared ($\eta_{\text{G}}^{2}$) was used for comparison of effect sizes between experiments (Olejnik and Algina, 2003).

In case of paired samples and when the data could not be assumed to be normally distributed, instead of parametric paired *t*-tests we used non-parametric equivalent Wilcoxon signed-rank tests.

## Results

For a first comparison with our previous experiment (Jacquey et al., 2020b) we analyzed the infant’s activity at successive 1-min intervals, expecting that, as was the case in our previous experiment, in the Contingent condition this activity would gradually increase over the course of the experiment, and would do so faster than in the Non-contingent condition. We also expected that, as in our previous experiment, if infants were able to detect the contingency and properly differentiate their arms, then activity of the Connected arm should be higher and/or increase faster compared to the Unconnected arm. Additionally, given the modifications to the paradigm that we were using as compared to our previous study, we hoped that all these effects would be stronger than in Jacquey et al. (2020b) as measured by generalized eta-squared ($\eta_{\text{G}}^{2}$) values.

### Results of the Present Experiment

**FIGURE 2:**
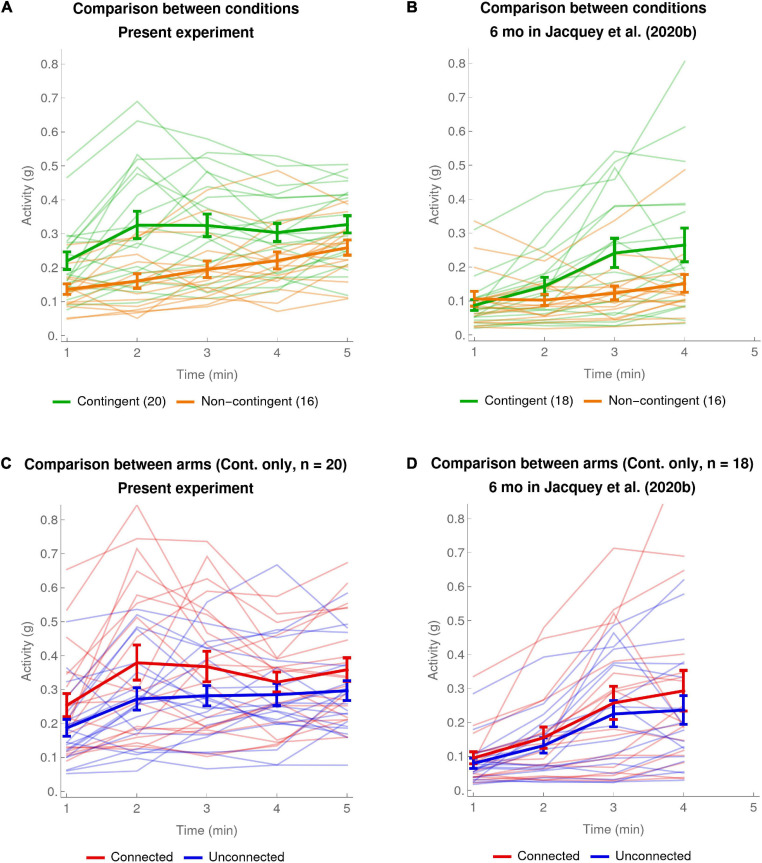
Average and individual per-minute activity. **(A,B)** Comparison between conditions. The thick green curve shows the mean of the pooled activity of both arms of the Contingent condition. The thick orange curve shows the same data for the Non-contingent condition. The error bars are one SEM above and below the mean. Thinner pale curves show individual data. **(A)** Present experiment: data of 20 infants in the Contingent condition and 16 infants in the Non-contingent condition (one far outlier excluded). **(B)** Data of 6-month-olds in Jacquey et al. (2020b): 18 infants in the Contingent condition and 16 infants in the Non-contingent condition (one far outlier excluded). **(C,D)** Comparison between arms (Contingent condition only). The thick red curve shows mean ± SEM of the activity of the Connected arm in the Contingent condition. The thick blue curve shows the same data for the Unconnected arm in the Contingent condition. Thinner pale curves show individual data. **(C)** Present experiment: data of 20 infants in the Contingent condition. **(D)** 6-month-olds in Jacquey et al. (2020b): data of 18 infants in the Contingent condition.

Figure 2A shows, separately for the Contingent and Non-contingent conditions, the pooled activity of both arms during the course of the experiment on a minute-by-minute basis, individually for all infants, with the means across infants shown as thicker lines. A mixed ANOVA, with Period of the experiment (minute-1 to minute-5 means) as a within-subject factor and Condition (Contingent versus Non-contingent) as a between-subject factor, showed that the main effects of both Condition and Period were statistically significant [Condition: *F*_(__1_,_34__)_ = 9.61, *p* < 0.004, generalized eta-squared $\eta_{\text{G}}^{2}$ = 0.17; Period: *F*_(__2_._6_,_87_._6__)_ = 13.81, with effective degrees of freedom Greenhouse–Geisser corrected, *p* < 0.001, $\eta_{\text{G}}^{2}$ = 0.1]. The Condition × Period interaction was also statistically significant [*F*_(__2_._6_,_87_._6__)_ = 3.08, Greenhouse–Geisser corrected, *p* < 0.05, $\eta_{\text{G}}^{2}$ = 0.02]. This analysis was done excluding one far outlier from the Non-contingent condition; the outlying value of that infant was also excluded from further analyses. (Note that an ANOVA including the outlying value led to the same conclusions apart from the Condition × Period interaction becoming marginally significant after Greenhouse–Geisser correction: *p* = 0.054). *Post hoc* tests done to further understand the Condition × Period interaction, revealed that the difference of activity between the two conditions was only significant for the second and third minute of the experiment [*t*_(__34__)_ = 3.62, *p* < 0.006 and *t*_(__34__)_ = 3.11, *p* < 0.02, two-sided and Bonferroni-corrected for 5 comparisons].

The significant main effect of contingency in the ANOVA is visible in Figure 2A from the fact that the curve for the Contingent condition is systematically higher than the curve for the Non-contingent condition (mean ± SEM for Contingent condition: 0.301 ± 0.026; mean ± SEM for Non-contingent condition: 0.195 ± 0.019 or 0.217 ± 0.028 including the one far outlier). The main effect of Period is visible from the fact that both curves rise somewhat over time. The Condition × Period interaction is visible in Figure 2A from the fact that the evolution of activity over time has different patterns for the two conditions. In the Non-contingent condition there is a gradual and fairly linear increase in pooled arm activity over the course of the experiment. In the Contingent condition the increase is less linear: activity increases during the first 2 min, and then plateaus toward the end of the experiment.

To verify that the unequal number of subjects (Contingent condition: 20; Non-contingent condition: 16) does not bias the results, in the Supplementary Material (see section “Verification Requested by One Reviewer of the ANOVAs and Other Analyses With Unbalanced Design”) we redid all the statistical analyses and data plots described above by randomly selecting 16 infants in the Contingent condition. We again obtained a significant effect of Condition, but the Condition × Period interaction was not significant. Again the curve for the Contingent group separates from the Non-contingent group during the very first minutes of the experiments and then reaches a plateau. Again, *t*-tests suggested that there is an activity difference between the two conditions only for the second and third minutes of the experiments.

Figure 2C shows a comparison of the activity of the Connected and Unconnected arms in the Contingent condition on a minute-by-minute basis. A mixed ANOVA, with experimental time Period (minute-1 to minute-5 means) and Arm (Connected versus Unconnected) as within-subject factors and connection Configuration (connected bracelet on the Right or the Left arm) as a between-subject factor, showed that the only statistically significant effects were the main effects of Arm and Period [Arm: *F*_(__1_,_18__)_ = 7.72, *p* < 0.013, generalized eta-squared $\eta_{\text{G}}^{2}$ = 0.05; Period: *F*_(__2_._3_, _41_._2__)_ = 6.47, Greenhouse–Geisser corrected, *p* < 0.003, $\eta_{\text{G}}^{2}$ = 0.07].

Compatible with the main effect of Arm in the ANOVA, Figure 2C shows that the Connected arm has a higher overall level of activity than the Unconnected arm (mean ± SEM of Connected arm: 0.337 ± 0.033; Unconnected arm 0.265 ± 0.025; mean difference: 0.072 ± 0.026); the main effect of Period is visible from the fact that both Connected and Unconnected arms increase their activity over the course of the experiment. In addition to what is shown by the analyses of variance, Figure 2C also suggests that both Connected and Unconnected arms increased their activity most quickly between the first and second minute of the experiment, peaking at the second and third minutes. This was to be expected given that the same was true of the pooled arm activity for the Contingent condition (see Figure 2A, thick green line and the *post hoc* tests for the second and third minutes of the Condition × Period interaction).

To summarize the results reported in this section, first we found clear sensitivity to the contingency over the whole experiment, as manifested by increased pooled arm activity in the Contingent condition as compared to the Non-contingent condition. This effect manifested itself by the fact that compared to the Non-contingent condition where activity rose gradually over the course of the experiment, in the Contingent condition arm activity increased abruptly between the first and the second minute, peaking in the second and third minutes. Second, we found a difference between the Connected and Unconnected arms, confirming sensitivity to contingency and indicating arm differentiation. This difference also seemed to start to appear very early, already in the first minute of the experiment.

### Comparison With Jacquey et al. (2020b)

Our hypothesis in this work was that the changes we made in the paradigm would make it easier for infants to detect the contingency as compared to Jacquey et al. (2020b). Fortunately we had available the raw data of the original experiment of Jacquey et al. (2020b), and were thus able to use it for a re-analysis. In order to make the comparison, we ran the same analyses on the same age group from Jacquey et al. (2020b), using the same pre-processing of acceleration values that we used for our own data (see section “Acceleration Data Processing”). This data included 18 infants in Jacquey et al.’s (2020b) Contingent condition and 16 infants in their Non-contingent condition (the data of the 17th infant was a far outlier and was excluded). Note that in Jacquey et al. (2020b), 4.5-s duration attention getters displayed at the beginning of every minute startled infants and caused their activity to briefly drop sharply. Therefore, like Jacquey et al. (2020b) did in their own analysis, we excluded the corresponding data.

Since our purpose in this section is to compare the results of our experiment with those of Jacquey et al. (2020b), an obvious first step is to perform an ANOVA on the data of the two experiments combined. Since our experiment lasted 5 min, whereas Jacquey et al.’s (2020b) lasted 4 min, we excluded the 5th minute from our experiment in the comparison. The mixed ANOVA with Period of the experiment (minute-1 to minute-4 means) as a within-subject factor and experimental Study [Jacquey et al.’s (2020b) versus the present study] and Condition (Contingent versus Non-contingent) as between-subject factors showed the following statistically significant effects: the main effect of Study [*F*_(__1_,_66__)_ = 10.33, *p* = 0.002, generalized eta-squared $\eta_{\text{G}}^{2}$ = 0.11]; the main effect of Condition [*F*_(__1_,_66__)_ = 11.85, *p* = 0.001, $\eta_{\text{G}}^{2}$ = 0.12]; the main effect of Period [*F*_(__2_._49_,_164_._2__)_ = 26.64, with effective degrees of freedom Greenhouse–Geisser corrected, *p* < 0.001, $\eta_{\text{G}}^{2}$ = 0.09]; the effect of the first-order interaction Condition × Period [*F*_(__2_._49_,_164_._2__)_ = 5.42, *p* = 0.003, Greenhouse–Geisser corrected, $\eta_{\text{G}}^{2}$ = 0.02]; the effect of the second-order interaction Study × Condition × Period [*F*_(__2_._49_,_164_._2__)_ = 4.76, Greenhouse–Geisser corrected, *p* = 0.006, $\eta_{\text{G}}^{2}$ = 0.02].

In order to further understand these effects, the following discussion additionally provides a comparison with the analyses we performed on our own data.

Figure 2B shows Jacquey et al.’s (2020b) results for the Contingent and Non-contingent conditions on a minute-by-minute basis. A mixed ANOVA, with Period of the experiment (minute-1 to minute-4 means) as a within-subject factor and Condition (Contingent versus Non-contingent) as a between-subject factor showed that the main effect of Condition was not statistically significant [Condition: *F*_(__1_,_32__)_ = 2.84, *p* = 0.101, generalized eta-squared $\eta_{\text{G}}^{2}$ = 0.06; or, including outlier: *F*_(__1_,_33__)_ = 0.8, *p* = 0.377, $\eta_{\text{G}}^{2}$ = 0.02]. The main effect of Period was statistically significant [Period: *F*_(__2_._02_,_64_._5__)_ = 17.16, Greenhouse–Geisser corrected, *p* < 0.001, $\eta_{\text{G}}^{2}$ = 0.12; or, including outlier: *F*_(__2_._35_,_77_._6__)_ = 16.61, Greenhouse–Geisser corrected, *p* < 0.001, $\eta_{\text{G}}^{2}$ = 0.09]. The effect of Condition × Period interaction was also statistically significant [*F*_(__2_._02_,_64_._5__)_ = 6.9, Greenhouse–Geisser corrected, *p* < 0.01, $\eta_{\text{G}}^{2}$ = 0.05; or, including outlier: *F*_(__2_._35_,_77_._6__)_ = 5.78, Greenhouse–Geisser corrected, *p* < 0.01, $\eta_{\text{G}}^{2}$ = 0.04]. To interpret the statistically significant interaction we compared the average activities in the two conditions for each of the 4 min. These *post hoc* tests showed that the difference of activity between the two conditions was significant for the third minute of the experiment [*t*_(__24_._1__)_ = 2.48, *p* < 0.05 Bonferroni-corrected for 4 comparisons, Satterthwaite approximation to the 32 effective degrees of freedom]. These results are compatible with what we see in Figure 2B, namely that activity in the Non-contingent condition hardly increased at all over the course of the experiment, whereas activity in the Contingent condition increased gradually with a clear difference appearing at the end, particularly in the third period.

Figure 2D shows the activity of Jacquey et al.’s (2020b) 6-month-olds’ Connected versus Unconnected arms on a minute-by-minute basis. A mixed ANOVA with experimental time Period (minute-1 to minute-4 means) and Arm (Connected versus Unconnected) as within-subject factors and connection Configuration (connected bracelet on the Right or the Left arm) as a between-subject factor, showed that only the Period has a statistically significant effect [Period: *F*_(__1_._97_,_31_._6__)_ = 15.56, Greenhouse–Geisser corrected, *p* < 0.001, $\eta_{\text{G}}^{2}$ = 0.18]. This effect of Period is visible in Figure 2D as a rising trend in activity for both arms over the course of the experiment. Importantly, the small difference visible between the activity of the Connected arm and the Unconnected arm was not statistically significant, nor were any interactions with the other factors.

In summary for this comparison section, a first point is that the comparison of our results with those of Jacquey et al. (2020b) shows that in our results the overall activity difference between Contingent versus Non-contingent conditions was statistically significant (effect size: $\eta_{\text{G}}^{2}$ = 0.17), whereas it was not in Jacquey et al. (2020b). A second point is that whereas in our experiment the *post hoc* tests showed that the effect of Condition was significant both in the second and third minute of the experiment, in Jacquey et al. (2020b) it was significant only in the third minute – suggesting that in our experiment the effect of contingency manifested itself earlier. A third point is that we find a main effect of arm differentiation, with the Connected arm moving more than the Unconnected arm (effect size: $\eta_{\text{G}}^{2}$ = 0.05), whereas our re-analysis of Jacquey et al. (2020b) showed that the effect of Arm, while descriptively present and visible in Figure 2D, was not strong enough to reach statistical significance.

These facts already partially confirm our hypothesis that our changes to the paradigm helped infants detect the contingency as compared to Jacquey et al. (2020b). What is most interesting, shown in Figures 2A,B and supported by the *post hoc* tests, is that in the present experiment the contingency effects appeared in the first few minutes of the experiment, and then seemed to level off after the third minute, whereas in Jacquey et al. (2020b) effects continued to build up more gradually. It would seem that in our experiment infants became aware of the existence of a contingency and increased their activity more quickly than in Jacquey et al. (2020b), but then subsequently no longer increased their activity.

### Evolution of Activity With Finer Temporal Resolution

The non-linearity in the shape of the curves for the data in the present experiment, with the largest change in activity in the Contingent condition seeming to occur between the first and second minutes (see Figures 2A,C), suggests that we should analyze the data at a finer temporal resolution.

We therefore decided to take the means over successive 10-s bins of activity instead of using 1-min bins as we had done in Jacquey et al. (2020b) and as is usually done in the literature on the mobile paradigm.

#### Comparison of Contingency Detection at 10-s Resolution for the Two Experiments

**FIGURE 3:**
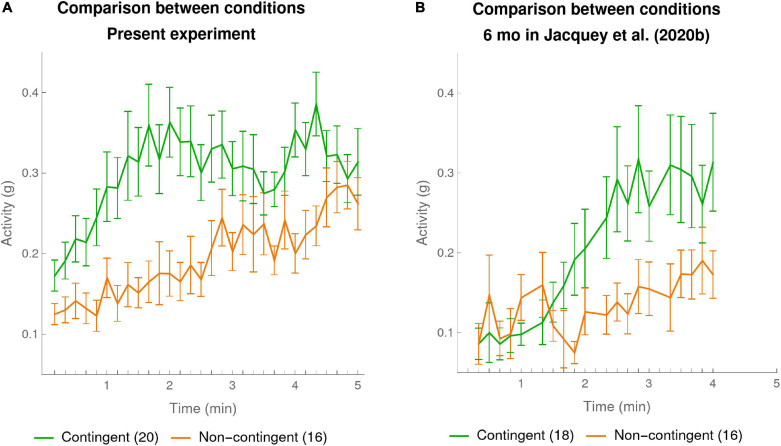
Mean activity per 10-s bin in each condition. The mean activities were calculated for every 10-s bin over the duration of experiment. The green curve shows the means of the participants in the Contingent condition. The orange curve shows data for the participants in the Non-contingent condition. The error bars are one SEM above and below the mean. **(A)** Present experiment: data of 20 infants in the Contingent condition and 16 in the Non-contingent condition (one far outlier excluded). **(B)** 6-month-olds in Jacquey et al. (2020b): data of 18 infants in the Contingent condition and 16 in the Non-contingent condition (one far outlier excluded).

First consider the plot in Figure 3A of infants’ activity for the present experiment, taken at a 10-s time resolution. The graph confirms what we had already shown in our analysis at the 1-min resolution, namely that the infants in the Contingent condition moved their arms significantly more than those in the Non-contingent condition. But the finer temporal resolution allows us to see that this effect was due to a very clear and sudden rise in activity of the Contingent condition starting at the beginning of the first minute of the experiment and continuing over 2 min, which then leveled off toward the end of the experiment. In comparison, in the Non-contingent condition the activity increased more gradually. (NB Supplementary Figure 3A shows the same trends when data analysis was restricted to a random subset of 16 of the 20 infants in the Non-contingent condition).

Figure 3B provides our re-analysis with 10-s bins of Jacquey et al.’s (2020b) group of 6-month-old infants [the data of other age groups in Jacquey et al. (2020b) is discussed in the Supplementary Material Section “10-s Resolution Reveals Effect of Age in Jacquey et al. (2020b)”]. Here also the finer temporal resolution revealed a fairly sudden rise of activity in the Contingent condition as compared to the Non-contingent condition. However, compared to the data in our own experiment, the moment when this happens appeared to come later, starting near the end of the second minute, and continuing to the end of the third minute.

To statistically compare this difference between the two experiments in the rate of increase of Contingent activities, we computed for each infant the slope of the infant’s activity over each of the first 4 min in the Contingent condition in the present experiment and in Jacquey et al.’s (2020b) experiment (linear regression over the 10-s bin values for each minute separately). The mean slope ± SEM for the first minute in the present experiment was 0.12 ± 0.05, significantly higher than 0.01 ± 0.04 in Jacquey et al. (2020b) though only before the (conservative) Bonferroni correction for 4 multiple comparisons [*t*_(__36__)_ = 1.86, *p* = 0.035 one-sided uncorrected for multiple comparisons]. None of the comparisons from minute 2 to 4 were statistically significant, even before correction for multiple comparisons. We thus have a degree of statistical confirmation of what is visible from Figures 3A,B, namely that activity in the Contingent condition of our present experiment started rising earlier, namely in the first minute, instead of starting to rise in the second minute in the corresponding condition of Jacquey et al. (2020b).

#### Early Contingency Detection Shown by 1-s Resolution

**FIGURE 4:**
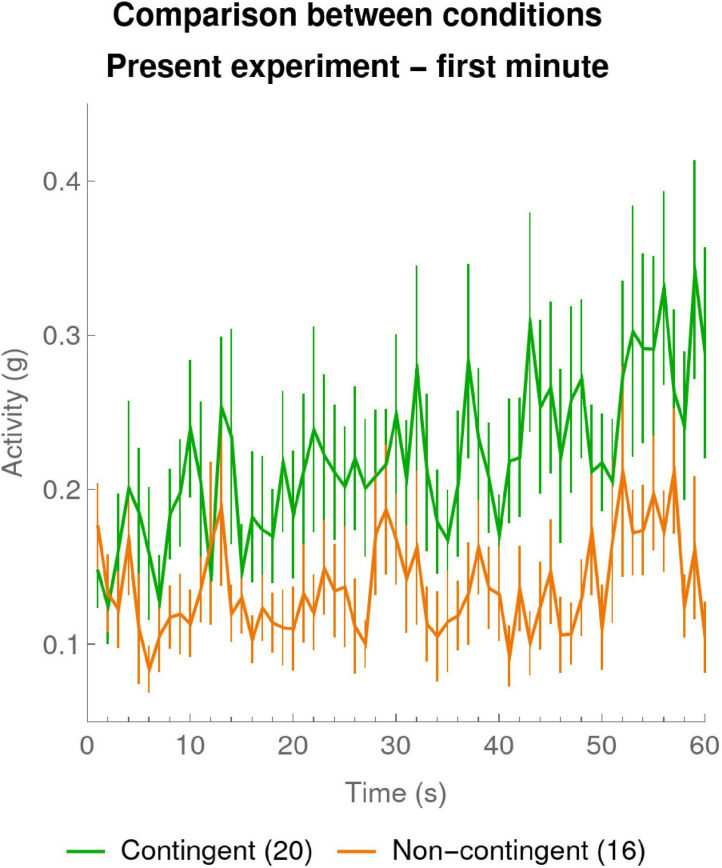
Zoom on mean activity during the first minute per 1-s bin. The mean activities were calculated for every 1-s bin over the first 60 s of the experiment. The green curve shows the means of the participants in the Contingent condition (data of 20 infants). The orange curve shows data for the Non-contingent condition, excluding one participant with outlying values (data of 16 infants). The error bars are one SEM above and below the mean.

It is worth pointing out a surprising aspect of the data of the present experiment in Figure 3A: already the very first data point, corresponding to the first 10 s of the experiment, seems to differ between the Contingent and Non-contingent conditions, suggesting that within 10 s of the beginning of our experiment, infants were able to detect the contingency. To further examine this effect in our experiment Figure 4 shows a “zoom” of the first minute of the experiment at 1-s resolution. At this very fine time scale we can already see evidence for a dissociation of the curves for Contingent versus Non-contingent activity. Note that in the first seconds of the experiment, activity rates for Contingent and Non-contingent conditions coincide. This is expected since at the beginning of the experiment infants cannot yet have discovered whether or not the stimulus is contingent on their actions.

To quantify this phenomenon we computed linear regressions for each individual participant for the 60 1-s bins of the first minute. In particular we wanted to verify that the activity in both conditions was initially at the same level as would be evidenced by similar *constants* in the regressions, but that the evolution of activity differed in the *slopes* of the regressions. The means ± SEM of the individual constants of the regressions were 0.16 ± 0.02 and 0.12 ± 0.01 for, respectively, the Contingent and the Non-contingent conditions and their difference was not statistically significant [*t*_(__32_._5__)_ = 1.557, *p* = 0.129 two-sided, Satterthwaite approximation to the 34 effective degrees of freedom]. The mean ± SEM of the individual slopes of the regressions were 0.002 ± 0.00007 and 0.0005 ± 0.0004, for, respectively, the Contingent and the Non-contingent conditions, and their difference was statistically significant [*t*_(__28_._2__)_ = 1.81, *p* < 0.05 one-sided, Satterthwaite approximation to the 34 effective degrees of freedom]. There was also a difference in overall total activity (mean ± SEM) over the whole 1-min period: the Contingent and Non-contingent activity were, respectively, 0.30 ± 0.03 and 0.19 ± 0.02, and the Contingent activity was significantly greater than the Non-contingent activity [*t*_(__32_._6__)_ = 3.25, *p* = 0.03 two-sided, Satterthwaite approximation to the 34 effective degrees of freedom]. This difference between Contingent and Non-contingent conditions was not statistically significant in the 1-min resolution analysis in Section “Results of the Present Experiment.”

The 1-s analysis of the first minute of the present experiment therefore confirms the surprising fact that already within seconds of the establishment of the contingency, infants became sensitive to it and start increasing their arm activity as compared to the Non-contingent condition.

#### Comparison of Arm Differentiation at 10-s Resolution for the Two Experiments

**FIGURE 5:**
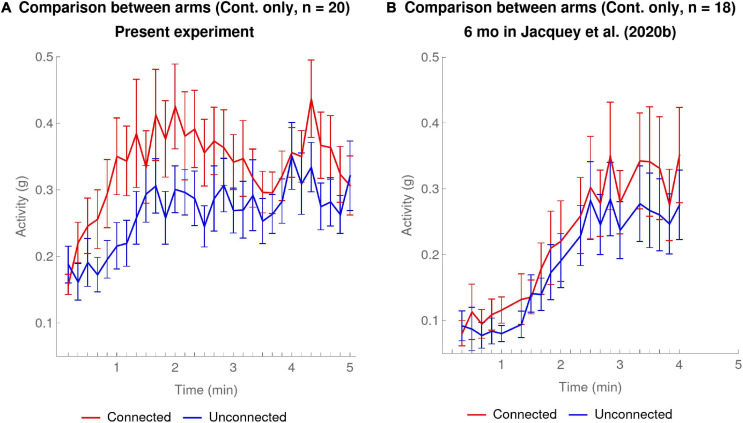
Contingent Condition only: mean activity at 10-s temporal resolution for Connected arm (red) versus Unconnected arm (blue). The error bars are one SEM above and below the mean. **(A)** Present experiment: data of 20 infants in the Contingent condition. **(B)** 6-month-olds in Jacquey et al. (2020b): data of 18 infants in the Contingent condition.

Figure 5A shows the results for arm differentiation at 10-s resolution for the present experiment. The ANOVA done on the 1-min resolution had already shown a significant effect of the Arm factor, visible here at 10-s resolution by the fact that the red curve corresponding to the Connected arm shows overall higher activation over the whole course of the experiment. What the higher temporal resolution now reveals is that this difference arose very quickly in the first minute of the experiment when the Connected arm activity rose faster than the Unconnected arm. To confirm this statistically, we computed the slopes in the same way as in Section “Comparison of Contingency Detection at 10-s Resolution for the Two Experiments” for each of the five 1-min periods of the experiment for the Connected arm and the Unconnected arm. Comparison of these activity slopes between arms revealed that there was a significant difference exclusively in the first minute, though only before the (conservative) Bonferroni correction for 5 multiple comparisons [paired *t*_(__19__)_ = 2.77, *p* = 0.012 one-sided], where the activity of the Connected arm increased significantly faster compared to the Unconnected arm.

Figure 5B shows the results for 6-month-olds in the experiment of Jacquey et al. (2020b). The red curve corresponding to the Connected arm has slightly higher activity than the blue curve, corresponding to the Unconnected arm. Despite the fact that our ANOVA re-analyzing Jacquey et al.’s (2020b) data at 1-min resolution had not shown an effect of Arm [see section “Comparison With Jacquey et al. (2020b)”], an additional analysis at the 10-s resolution including all the age groups in Jacquey et al. (2020b) detected a small but significant difference [see Supplementary Material Section “10-s Resolution Reveals Effect of Age in Jacquey et al. (2020b)”]. What the higher temporal resolution reveals here is that contrary to the present experiment, there was no moment over the course of the experiment where the Connected arm clearly separated away from the Unconnected arm. Indeed a comparison of activity slopes calculated for each minute as in Section “Comparison of Contingency Detection at 10-s Resolution for the Two Experiments” for the Connected versus Unconnected arms for the 4 min of the experiment revealed no significant differences, not even before the Bonferroni correction for 4 multiple comparisons (one-sided paired *t*-tests).

#### Conclusion on Fine Temporal Analysis

The fine temporal resolution analysis complements the conclusion from the 1-min analysis in an important way. The 1-min analysis had shown a greater effect size of contingency in the present experiment as compared to Jacquey et al. (2020b), and that the effect of arm differentiation was present for us, but not present for Jacquey et al. (2020b).

The 10-s resolution now additionally revealed that these differences were due to the fact that in the present experiment the contingency was discovered very quickly, within seconds of its establishment (as confirmed also by an additional 1-s resolution analysis), whereas this only seemed to start occurring at the end of the second minute in Jacquey et al. (2020b). In both experiments the curves suggest that this sudden increasing activity in the Contingent condition stopped and leveled off after about 1.5 min after its initiation. In the Non-contingent condition, in both experiments, activity rose gradually and continuously throughout the experiment, even reaching the same level as the Contingent condition in the present experiment. We assume that this gradual rise in activity in the Non-contingent condition corresponded to a gradual increase in global excitation on the part of the infant, and that it was probably also present in the Contingent condition but not visible because it was masked by the strong increase occurring there due to the discovery of the contingency. The reason why in Jacquey et al. (2020b) Non-contingent activity never reached the level of the Contingent activity could be because the rate of increase was in any case slower in that experiment, and because that experiment only lasted 4 min.

As concerns arm differentiation (Connected versus Unconnected) in the Contingent condition, the 10-s resolution analysis also provides valuable insights as compared to the 1-min analysis. In the present experiment, where the 1-min resolution analysis had shown a significant effect of Arm, the 10-s resolution revealed the moment that the effect became manifest, namely within the very first minute of the experiment. Furthermore this preferential activation of the Connected arm seemed to plateau after about 2 or 3 min. The conclusion seems to be that infants detected which of their two arms was the Connected arm almost as soon as they detected the contingency, but possibly lost interest after about 2–3 min.

In Jacquey et al. (2020b) the 1-min resolution analysis had not shown a significant effect of Arm at 6 months, but the 10-s analysis suggested a small overall main effect. On the other hand this fine resolution analysis revealed an important difference with our experiment, namely that there was no clear moment when the slope of the Connected arm became clearly greater than that of the Unconnected arm. It would seem that differentiation between the two arms therefore occurred more gradually than in the present experiment.

A final point worth noting about the advantage of 10-s resolution analysis concerns a reanalysis that we did of Jacquey et al. (2020b)’s 4- and 8-month-old’s data in the Supplementary Material [see section “10-s Resolution Reveals Effect of Age in Jacquey et al. (2020b)”]. These authors had curiously not found an effect of age either on contingency detection, nor on arm differentiation. Our 10-s analysis, however, revealed that in fact there was a gradually increasing effect of arm differentiation with age. We argue in the Supplementary Material that it may precisely be infants’ ability to do arm differentiation at 8 months that explains why *pooled* arm activity at 8 months no longer seemed sensitive to the contingency, since the contingent arm would have been moving more and the non-contingent arm would have been moving less. This explains why there appeared to be no effect of age on contingency. It was masked by greater ability to do arm differentiation in the oldest age group.

### Two Alternative Measures of Contingency Detection

We have seen in the previous sections that analysis of arm activity at 10-s resolution revealed fast responses that were not visible in analyses at 1-min resolution. It seems that infants’ reaction to the contingency was not simply to gradually increase their activity. On the contrary, the present data suggest that their reaction to the contingency was much quicker than usually thought, but may also have faded out in a matter of minutes. Another, not incompatible possibility mentioned in the introduction has also been suggested by Watson (1979, 1984, 1985), and Movellan (2005) and Butko and Movellan (2010). In this, when confronted with a contingency, an infant will want to explore the contingency by modulating its activity in order to determine if the infant itself is really the source of the stimulation. We here present two additional measures of behavior that might detect such exploratory activity, and that might be interesting to use in future work on contingency detection.

#### Repeating Motions That Provoke a Stimulation

As mentioned in the introduction, Watson (1984) had suggested that when an infant triggers a stimulus by making a movement of one of its limbs, the infant may try to reproduce the stimulation by repeating the movement that it just made. To evaluate this hypothesis in our data, we compared the activity immediately following a sensory stimulus triggered by a motor response with the activity during other moments of the experiment.

##### Motion spikes following stimulus triggering

We defined a “window of interest” following a stimulus occurrence where we considered that a modification of the infant’s behavior was likely to occur. This window, we assumed, would coincide with the time over which the infant’s attention span would suffice to attend to the events following lighting up of the bracelets, i.e., to the response–stimulus pair. Our window of interest consisted of 13 successive 1-s intervals following the occurrence of the stimulus [Watson (1984) also used 13 successive 1-s intervals, except he smoothed activity with a 3-s running average].

Following Watson (1984), we expected that during the window of interest the infants in the Contingent condition might try to repeat the sharp arm movement that triggered the stimulus—we will call such sharp movements *motion spikes*. For each infant, we defined a motion spike as an arm movement with an acceleration greater than the third quartile of the distribution of joint activity of both arms throughout the experiment. This definition of motion spikes relative to an individual infant’s global activity distribution allowed us to detect motion spikes equally well for infants with high or low average activity levels.

We divided each 13-s window of interest following a stimulus-triggering moment into 1-s bins and computed the proportion of data samples in the bin that corresponded to motion spikes. We will illustrate this computation with an example. If for an infant, there are 10 response–stimulus pairs over the whole experiment, there will be 10 windows of interest. The first bin of each window of interest corresponds to the time immediately after the end of the stimulus up to 1 s after it. We computed the proportion of motion spikes in this first bin for each of the 10 windows of interest and averaged these proportions to get the mean proportion of motion spikes in this first bin of the windows of interest. We called this the value “inside bin.” This value then had to be compared to the expected proportion of motion spikes that would have occurred in the bin if it had not been preceded by a stimulus. To estimate this, we calculated the proportion of motion spikes that occurred over the whole experiment *except these particular 1-s intervals following a stimulus in all the windows of interest*. We call this the proportion “outside bin” (note that the proportion *outside bin* for each particular bin is a potentially conservative estimate since it includes, besides data samples actually *outside* the windows of interest, the other 12 remaining bins *within* windows of interest). This whole process was repeated for the second 1-s bins, third 1-s bins, etc. up to the thirteenth bins. We thus finally obtained two curves, one curve corresponding to the proportions of motion spikes at 13 moments *inside* the bins of the window of interest following a stimulus trigger, and the other curve corresponding to the proportions of motion spikes *outside* each of the bins of such windows of interest. If the frequency of motion spikes in the window of interest is related to the response–stimulus contingency, then in the Contingent condition in which such a contingency is present, the frequencies observed in the windows of interest should be higher than the frequencies observed outside them.

In the Non-contingent condition the motion spikes were computed in the same way but they corresponded to the infant’s behavior following a stimulus only, instead of to a response–stimulus pair. If the frequency of motion spikes in the window of interest is related to the response–stimulus contingency, then in the Non-contingent condition in which such a contingency is absent, the frequency observed in the windows of interest should not differ from the frequencies observed outside them.

Watson (1984) noted that the frequency increase inside the window of interest could also be due to the infant reacting to the stimulation only, which he calls “reaction to the stimulus.” The comparison between Contingent and Non-contingent conditions should allow to rule out that the infants are reacting to the stimulus only because the two conditions differ only in the presence or absence of the contingency; specifically, if the increase (of motor spike proportion inside versus outside the window of interest) in the Contingent condition is higher than the increase in the Non-contingent condition, the cause can be attributed to the response–stimulus contingency, that is, to the reaction to the response–stimulus pairing.

Watson (1984) also examined another possible cause of activity increase inside the window of interest, namely that infants could be reacting to their own body movements, by repeating them in some way—he calls this “reaction to having responded.” In the present experiment, comparison between the Connected arm and the Unconnected arm activity also provides a way to test this aspect; in particular, if the increase of motor spike proportion inside versus outside the window of interest in the Connected arm is higher than in the Unconnected arm, the cause should be the triggering of the response–stimulus contingency by the former but not the latter, thus ruling out that infants were only reacting to their own previous arm movements.

##### Motion spikes in Contingent versus Non-contingent conditions

**FIGURE 6:**
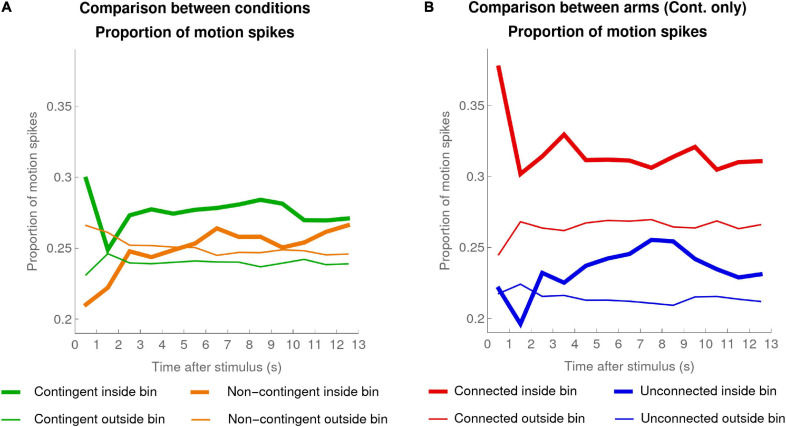
Proportion of motion spikes. **(A)** Mean (over the individual means) proportion of motion spikes for pooled activity of both arms for each infant in the Contingent condition (green, data of 20 infants) and the Non-contingent condition (orange, data of 16 infants), computed for the 13 1-s bins inside windows of interest (thick lines) and outside the windows of interest (thin lines). **(B)** Contingent Condition only: mean (over the individual means) proportion of motion spikes for the Connected (red) and Unconnected (blue) arms for infants, inside (thick lines) and outside (thin lines) the windows of interest.

Figure 6A shows the mean taken over all infants of individual mean proportions of motion spikes, inside and outside windows of interest for each condition.

In the Contingent condition, the high values during the first second after a stimulus activation probably reflect motor inertia following the triggering of the stimulus (i.e., the arm motion that triggered the stimulus continues for a short time after the stimulus has been triggered). To reduce the influence of this motor inertia, we excluded the data of the first bin when we compared the average activity inside and outside the window of interest. Note that this exclusion goes against our hypothesis. In the Contingent condition, the comparison revealed that there were significantly more motion spikes inside compared to outside the window of interest (Wilcoxon signed-rank test, test statistic = 199, *p* < 0.0005). Analyzing the data of the Non-contingent condition in exactly the same way revealed no significant difference inside versus outside the window of interest (Wilcoxon signed-rank test, test statistic = 92, *p* = 0.224). Additionally, the mean difference (mean ± SEM) in the proportion of motion spikes inside versus outside the window of interest was larger in the Contingent condition (0.03 ± 0.007) compared to the Non-contingent condition (0.003 ± 0.003); *t*_(__27_._95__)_ = 4.03, *p* < 0.0005 two-sided, Satterthwaite approximation to the 34 effective degrees of freedom.

The fact that the proportion of motion spikes inside the window of interest was significantly higher than the proportion outside it but only in the Contingent condition, together with the fact that the mean difference in proportion (inside - outside) was higher in the Contingent condition compared to the Non-contingent condition, suggest that infants were not merely reacting to the stimulus but were indeed sensitive to the contingency.

##### Motions spikes in Connected versus Unconnected arms

The question now further arises of whether infants were not just indiscriminately moving both arms to attempt to replicate the effects of their actions, but were also trying to determine which of their two arms triggered the occurrence of the stimulus. If the latter is true, and if the infants were not merely reacting to having responded (i.e., reacting to their own arm movements by repeating them), then we would expect that in the window of interest following a stimulus appearance the proportion of subsequent motion spikes observed would be higher for the Connected arm than for the Unconnected arm.

Figure 6B shows the proportion of motion spikes inside and outside the window of interest, separately for the Connected and the Unconnected arms. The mean difference of motion spike proportions inside versus outside the window of interest was significantly positive for the Connected arm (mean difference: 0.05; Wilcoxon signed-rank test, test statistic = 198, *p* < 0.001 one-sided) but also for the Unconnected arm (mean difference: 0.02; Wilcoxon signed-rank test, test statistic = 171, *p* < 0.01 one-sided). While both Connected and Unconnected arm mean differences were significantly positive, the mean difference in the Connected arm was larger compared to the Unconnected arm (Wilcoxon signed-rank test, test statistic = 36, *p* < 0.05 two-sided).

In sum, both Connected and Unconnected arms increased their number of motion spikes inside the window of interest compared to outside the window of interest, but this increase was larger for the Connected arm. The larger increase for the Connected arm is not compatible with the interpretation that the infants reacted to their own arm movements and confirms that the infants were indeed reacting to the contingency.

Our analyses in this section confirm Watson’s (1984) suggestion that infants tended to reproduce a movement that had just previously triggered the occurrence of a stimulus. More work could be done concerning the size of the window of interest and concerning other possible conditional probability measures, but these findings are a first promising step in the direction of Watson’s (1984) suggestions.

#### Extreme Values in the Acceleration Distribution

A second idea about what an infant might do to test whether the infant itself is at the origin of the changes in stimulation that are occurring in its environment was suggested by Movellan (2005) and Butko and Movellan (2010). The idea is that infants could alternate periods of moving and freezing in order to check the degree of link between their actions and the occurrence of a stimulation. If it is true that when confronted with a contingency infants exhibit such stop-and-go activity, one would expect that the incidence of both very small accelerations (corresponding to stops) and very large accelerations (corresponding to goes) would be greater in the Contingent as compared to Non-contingent conditions. One way to check for this is to look at the lower and upper deciles of the acceleration distributions. In the Contingent condition these deciles should be pushed toward the extremes compared to the lower and upper deciles observed in the Non-contingent condition.

##### Time evolution of deciles in Contingent versus Non-contingent conditions

**FIGURE 7:**
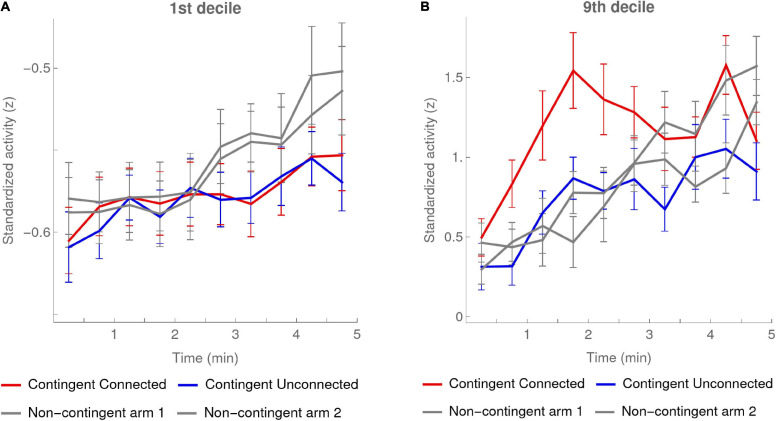
1st and 9th deciles per 30-s bin. Activity deciles were calculated for every 30-s bin over the first 5 min of the experiment. The red and blue curves show the means of the participants in the Contingent condition (data of 20 infants) for, respectively, the activity of the Connected and the Unconnected arm. The error bars are one SEM above and below the mean. The gray curves show the same data for each arm activity in the Non-contingent condition, excluding the one participant with outlying values (data of 16 infants). In the Non-contingent condition the categories “arm-1” and “arm-2” were randomly attributed while approximately counterbalancing the number of left and right arms in each class. **(A)** 1st decile. **(B)** 9th decile.

Figure 7 shows the evolution of the first and the ninth deciles during the experiment in each condition. To calculate these deciles we standardized the activity values for each participant separately based on the mean and standard deviation of the pooled activity of both arms, and used *z*-scores throughout this analysis. In order to have enough values in each time bin, we used larger, 30-s bins.

Supporting our predictions, the value of the first decile in the Contingent condition increased about two times slower than in the Non-contingent condition (Figure 7A). The slopes (mean ± SEM) of the linear regressions of the pooled arm activity on time periods of 30 s in the Contingent and Non-contingent conditions were, respectively, 0.005 ± 0.001 and 0.009 ± 0.001; the mean slope in the Contingent condition was significantly smaller than the slope in the Non-contingent condition [*t*_(__34__)_ = −2.16, *p* < 0.5 two-sided].

Importantly, note that this slower increase in the first decile in the Contingent condition as compared to the Non-contingent condition occurred even though the *mean* of the Contingent condition increased *faster* than that of the Non-contingent condition (see Figures 3A, 5A). This counterintuitive finding lends further credence to the validity of Movellan’s (2005) hypothesis.

The values of the 9th deciles tell a complementary story. As seen from the plot in Figure 7B, these rose gradually over the course of the experiment except for the Connected arm, for which, on the other hand, they rose very suddenly from the very beginning of the experiment, and then reached a plateau at the second minute. This observation from the graph was confirmed by linear regressions we performed on each half of the experiment for each condition. These showed significantly different slopes when comparing the first and second half of the experiment only f or the Connected arm: the slopes were: (mean ± SEM) 0.24 ± 0.06 and 0.01 ± 0.05 and their difference of 0.23 ± 0.089 was significant [paired *t*_(__19__)_ = 2.64, *p* < 0.5 two-sided]. This result is analogous to what we had observed for the mean activity shown in Figure 5A, where there was a very sharp rise in activity of the Connected arm as compared to the Unconnected arm. In the Non-contingent condition, on the other hand, there were no differences in the slopes between first and second halves of the experiment for either arm.

When considered together, these observations about the 1st and 9th deciles confirm Movellan’s (2005) suggestion of a stop-and-go behavior, with the stops occurring for both arms and the goes only for the Connected arm. In the Non-contingent condition the entire distribution shifts to the right in a rather uniform way, possibly reflecting increased agitation of the infants during the experiment.

## Discussion

One of our aims in the present paper was to test whether, by using a more effective set of experimental conditions, we would be able to achieve more robust evidence of infants’ sensitivity to contingency in the laboratory than authors have found in the past, and in particular as compared to Jacquey et al. (2020b). Our results do indeed argue in favor of such greater sensitivity. We found a significant effect of contingency versus non-contingency, whereas in Jacquey et al. (2020b), with a similar number of infants, only the difference in slopes of the activity curves over time was significant. We also found a more rapid appearance of the effects of contingency and arm differentiation, starting as early as tens of seconds into the experiment. Finally we found larger generalized eta-squared values for our effects.

As proposed in our hypotheses presented in the Introduction, we suggest that our finding of greater sensitivity to the contingency in the present experiment as compared to Jacquey et al. (2020b) derives from the different modifications we made in our experimental paradigm.

First, our stimuli were binary with a high triggering threshold: when the amplitude of the infant’s movement passed this threshold the stimulus was triggered, but the intensity of the stimulus was fixed. This ensured that a very clear, well-defined movement, easily recognizable by the infant could be associated with stimulus appearance. This is in contrast to Jacquey et al. (2020b), and more generally to the mobile paradigm, which is a “conjugate” paradigm where small limb movements can provoke small stimulation changes—meaning that the relation between movement and stimulation is less clear-cut.

Second, our stimuli were localized on bracelets that were placed on the infants’ wrists, that is, in the infants’ proximal space, while in Jacquey et al. (2020b) the stimuli appeared on a computer display out of the infants’ reach, in their distal space. Infants around age 4–8 months may be particularly occupied with learning to control their own limbs, and so such a proximal stimulation may be of more interest to them than a distal stimulation (Rochat and Goubet, 1995; Rochat et al., 1999).

Third, stimuli and effectors were coincident and spatially lateralized, making it easy to determine the origin of the contingency. Again this is an aspect of our contingency that is similar to the type of limb-related contingencies that infants may be probing at this age.

Finally, because of their weight on the infants’ wrists, our bracelets provided some degree of proprioceptive feedback when infants moved their arms, something the bracelets in the previous study lacked, but that was shared with the ribbon used in the mobile paradigm—since it presumably offered some resistance when the infant pulled on it.

All of these factors may have contributed to increasing the ease with which our infants detected the contingency. Further work will be able to investigate which of these different manipulations were the most effective.

Perhaps more interesting and important than the greater sensitivity to the contingency that we have demonstrated here is the fact that our work prompted us to re-analyze our data, and also those of Jacquey et al. (2020b), at a finer temporal resolution of 10 s rather than the usual 1-min resolution used by us previously and in the literature. This reanalysis revealed that infants at 6 months were surprisingly rapid in discovering the contingency, showing a sudden increase in arm activity within tens of seconds compared to the Non-contingent condition. Not only were 6-month-old infants able to quickly detect a contingency as compared to a Non-contingent condition, but they were also able to quickly localize the origin of the contingency to one or other arm.

As shown in the Supplementary Material (see section “10-s Resolution Reveals Effect of Age in Jacquey et al. (2020b)”) it is worth pointing out that our finer temporal analysis also allowed us to cast some light on why Jacquey et al. (2020b) found that infants do not increase their ability to detect a contingency as they get older. Our 10-s analysis suggests that in fact infants were perfectly able to detect the contingency, and did so better as they grew older. In particular, the oldest group was also able to very well distinguish the arm that caused the stimulation. This had the consequence that they moved the Connected arm *more* and the Unconnected arm *less.* The pooled arm activity therefore did not increase as much as would have been expected in the oldest group. This explains why Jacquey et al. (2020b) found an apparent lack of effect of age on contingency detection.

A major contribution of our paper is therefore the suggestion that examination of changes in limb activity at a finer temporal scale than is usually done in the literature may reveal phenomena that are masked at 1-min or longer resolutions. In particular, both in our experiment and that of Jacquey et al. (2020b), the time course of limb activity is surprisingly fast, and dies out after less than 2 min. It may be that up till now researchers have underestimated infants’ ability to detect a contingency in the laboratory because analysis at a coarse temporal resolution fails to pick up very fast reactions that die out quickly and may be followed by boredom and fussiness.

Another contribution of our paper is our demonstration of the potential usefulness of two additional measures of sensitivity to contingency. Both measures are motivated by the idea suggested by Watson (1979, 1984, 1985) and Movellan (2005) according to which, in the presence of a contingency, infants will tend to explore that contingency with the aim of determining if they are actually the cause of the effects it is producing.

One consequence of this idea is the proposal by Watson (1984) that the probability that an infant should repeat an action that has produced a stimulation should be higher than the probability of making that action without being preceded by a stimulation. Our investigation of that idea indeed confirmed this to be the case in a time window of 13 s after a stimulation. Further work could refine this finding and potentially exploit it as a measure of sensitivity to contingencies.

Another consequence of the idea that infants should try to explore a contingency was suggested by Movellan (2005) and Butko and Movellan (2010). According to this, a good way for an infant to determine if it is the origin of a stimulation is for the infant to adopt a stop-and-go strategy where it sometimes acts, and sometimes “freezes” in order to test whether the stimulation occurs without the infant moving. We checked a prediction of this hypothesis, which is that there should be evidence of more stopping, and more large movements, in the contingent condition of our experiment. We did indeed find evidence supporting this, again suggesting that such measures may be exploited in future studies of contingency detection.

## Data Availability Statement

The raw data supporting the conclusions of this article are available from the authors on request.

## Ethics Statement

The studies involving human participants were reviewed and approved by University Paris Descartes. Written informed consent to participate in this study was provided by the participants’ legal guardian/next of kin.

## Author Contributions

SP and JO’R designed the experiments. SP and JV ran the subjects. SP and AD analyzed the data. SP and JO’R wrote the manuscript. JV and AD helped to improve the article. All authors contributed to the article and approved the submitted version.

## Conflict of Interest

The authors declare that the research was conducted in the absence of any commercial or financial relationships that could be construed as a potential conflict of interest.

## Footnotes

1 https://microbit.org

2 https://kitronik.co.uk

## References

[B1] Angulo-Kinzler R. M. Ulrich B. Thelen E. (2002). Three-Month-Old Infants Can Select Specific Leg Motor Solutions. *Motor Contr.* 6 52–68. 10.1123/mcj.6.1.52 11842270

[B2] Butko N. J. Movellan J. R. (2010). Detecting contingencies: An infomax approach. *Neural Netw. Offic. J. Int. Neural Netw. Soc.* 23 973–984. 10.1016/j.neunet.2010.09.001 20951334

[B3] Edelman G. M. (1987). *Neural Darwinism: The theory of neuronal group selection.* New York, NY: Basic Books, 371. 10.1126/science.240.4860.1802 17842436

[B4] Gibson E. J. (1988). Exploratory Behavior in the Development of Perceiving, Acting, and the Acquiring of Knowledge. *Annu. Rev. Psychol.* 39 1–42. 10.1146/annurev.ps.39.020188.000245

[B5] Jacquey L. (2019). *La sensibilité aux contingences sensorimotrices chez le bébé et son rôle dans le développement du savoir-faire corporel. Approche croisée en robotique et psychologie du développement.* Ph D. Thesis. Paris: Université de Paris.

[B6] Jacquey L. Fagard J. Esseily R. O’Regan J. K. (2020a). Detection of sensorimotor contingencies in infants before the age of 1 year: A comprehensive review. *Dev. Psychol.* 56 1233–1251. 10.1037/dev0000916 32463268

[B7] Jacquey L. Popescu S. T. Vergne J. Fagard J. Esseily R. O’Regan K. (2020b). Development of body knowledge as measured by arm differentiation in infants: From global to local? *Br. J. Dev. Psychol.* 38 108–124. 10.1111/bjdp.12309 31705684 PMC7065080

[B8] Kato M. Watanabe H. Taga G. (2013). Diversity and changeability of infant movements in a novel environment. *J. Motor Learning Dev.* 1 79–88. 10.1123/jmld.1.4.79

[B9] Keppel G. Wickens T. D. (2004). *Design & Analysis: A Researcher’s Handbook*, 4th Edn. London: Pearson.

[B10] Lawrence M. A. (2016). *ez: Easy Analysis and Visualization of Factorial Experiments* (Version 4.4-0). Vienna: R Core Team.

[B11] Lewis M. Sullivan M. W. Brooks-Gunn J. (1985). Emotional behaviour during the learning of a contingency in early infancy. *Br. J. Dev. Psychol.* 3 307–316. 10.1111/j.2044-835X.1985.tb00982.x

[B12] Merz E. C. McDonough L. Huang Y. L. Foss S. Werner E. Monk C. (2017). The mobile conjugate reinforcement paradigm in a lab setting. *Dev. Psychobiol.* 59 668–672. 10.1002/dev.21520 28436585 PMC5716629

[B13] Millar W. S. (1972). A Study of Operant Conditioning under Delayed Reinforcement in Early Infancy. *Monogr. Soc. Res. Child Dev.* 37 1–44. 10.2307/1165687 5073989

[B14] Millar W. S. (1975). Visual attention to contingent and non-contingent stimulation in six-and nine-month-old infants. *Psychol. Res.* 37 309–319. 10.1007/bf00309725 1187947

[B15] Millar W. S. Schaffer H. R. (1972). The influence of spatially displaced feedback on infant operant conditioning. *J. Exp. Child Psychol.* 14 442–453. 10.1016/0022-0965(72)90064-1 4658484

[B16] Movellan J. R. (2005). An Infomax Controller for Real Time Detection of Social Contingency. *Proc. 4th Int. Confer. Dev. Learning* 2005 19–24. 10.1109/DEVLRN.2005.1490937

[B17] Olejnik S. Algina J. (2003). Generalized Eta and Omega Squared Statistics: Measures of Effect Size for Some Common Research Designs. *Psychol. Methods* 8 434–447. 10.1037/1082-989X.8.4.434 14664681

[B18] Piaget J. (1936). *The origins of intelligence in children.* New York, NY: Basic Books.

[B19] R Core Team. (2020). *R: A Language and Environment for Statistical Computing (Version 4.0.3).* Vienna: R Core Team.

[B20] Rochat P. Goubet N. (1995). Development of sitting and reaching in 5- to 6-month-old infants. *Infant Behav. Dev.* 18 53–68. 10.1016/0163-6383(95)90007-1

[B21] Rochat P. Goubet N. Senders S. J. (1999). To reach or not to reach? Perception of body effectivities by young infants. *Infant Child Dev.* 8 129–148. 10.1002/(SICI)1522-7219(199909)8:3<129::AID-ICD193<3.0.CO;2-G

[B22] Rovee C. K. Rovee D. T. (1969). Conjugate reinforcement of infant expl oratory behavior. *J. Exp. Child Psychol.* 8 33–39. 10.1016/0022-0965(69)90025-3 5804591

[B23] Rovee-Collier C. K. Gekoski M. J. (1979). “The Economics of Infancy: A Review of Conjugate Reinforcement,” in *Advances in Child Development and Behavior*, Vol. 13 eds Reese H. W. Lipsitt L. P. (Amsterdam: Elsevier), 195–255. 10.1016/S0065-2407(08)60348-1 484323

[B24] Rovee-Collier C. K. Morrongiello B. A. Aron M. Kupersmidt J. (1978). Topographical response differentiation and reversal in 3-month-old infants. *Infant Behav. Dev.* 1 323–333. 10.1016/S0163-6383(78)80044-7

[B25] Sullivan M. W. Lewis M. (1989). Emotion and Cognition in Infancy: Facial Expressions during Contingency Learning. *Int. J. Behav. Dev.* 12 221–237. 10.1177/016502548901200206

[B26] Thelen E. (1995). Motor development: A new synthesis. *Am. Psychol.* 50 79–95. 10.1037/0003-066X.50.2.79 7879990

[B27] Watanabe H. Taga G. (2006). General to specific development of movement patterns and memory for contingency between actions and events in young infants. *Infant Behav. Dev.* 29 402–422. 10.1016/j.infbeh.2006.02.001 17138294

[B28] Watanabe H. Taga G. (2009). Flexibility in infant actions during arm- and leg-based learning in a mobile paradigm. *Infant Behav. Dev.* 32 79–90. 10.1016/j.infbeh.2008.10.003 19081637

[B29] Watanabe H. Taga G. (2011). Initial-state dependency of learning in young infants. *Hum. Movement Sci.* 30 125–142. 10.1016/j.humov.2010.07.003 21163544

[B30] Watanabe H. Homae F. Taga G. (2011). Developmental emergence of self-referential and inhibition mechanisms of body movements underling felicitous behaviors. *J. Exp. Psychol. Hum. Percept. Perfor.* 37 1157–1173. 10.1037/a0021936 21500942

[B31] Watson J. S. (1966). The development and generalization of “contingency awareness” in early infancy: Some hypotheses. *Merrill Palmer Quart. Behav. Dev.* 12 123–135.

[B32] Watson J. S. (1979). “Perception of Contingent as a Determinant of Social Responsiveness,” in *Origins of the infant’s social responsiveness*, ed. Thoman E. (New Jersey, NJ: Lawrence Erlbaum Associates), 33–64.

[B33] Watson J. S. (1984). “Bases of Causal Inference in Infancy: Time, Space and Sensory Relations,” in *Advances in Infancy Research*, Vol. 3 eds Lipsitt L. P. Rovee-Collier C. (New Jersey, NJ: Ablex), 152–165.

[B34] Watson J. S. (1985). “Contingency Perception in Early Social Development,” in *Social Perception in Infants*, eds Field T. M. Fox N. A. (New Jersey, NJ: Ablex), 157–176.

[B35] Watson J. S. Ramey C. (1969). *Reactions to response–contingent stimulation in early infancy.* Santa Monica, CA: Society for Research in Child Development.

[B36] Watson J. S. Ramey C. T. (1972). Reactions to response-contingent stimulation in early infancy. *Merrill Palmer Quart. Behav. Dev.* 18 219–227.

[B37] Zwicker S. Moore C. Povinelli D. J. (2012). “The development of body representations: the integration of visual-proprioceptive information,” in *Early development of body representations*, eds Brownell C. A. Slaughter V. (Cambridge: Cambridge University Press), 19–36.

