# Supplementary material for: 6-Month-Old Infants’ Sensitivity to Contingency in a Variant of the Mobile Paradigm With Proximal Stimulation Studied at Fine Temporal Resolution in the Laboratory
Source: Front Psychol. 2021 Mar 5;12:610002. doi: 10.3389/fpsyg.2021.610002 (PMC7973047; doi:10.3389/fpsyg.2021.610002)
Supplement: Supplementary file 1 [file Data_Sheet_1.pdf]

Supplementary material

S.1 Comparison between arms in the Non-Contingent condition

For the purpose of comparison, **Supplementary Figure 1** below is equivalent to **Figure 2B** of the "Results" section but instead includes infants in the Non-Contingent condition. In the Non-Contingent condition the categories "Arm 1" and "Arm 2" were randomly attributed while approximately counterbalancing the number of left and right arms in each class.

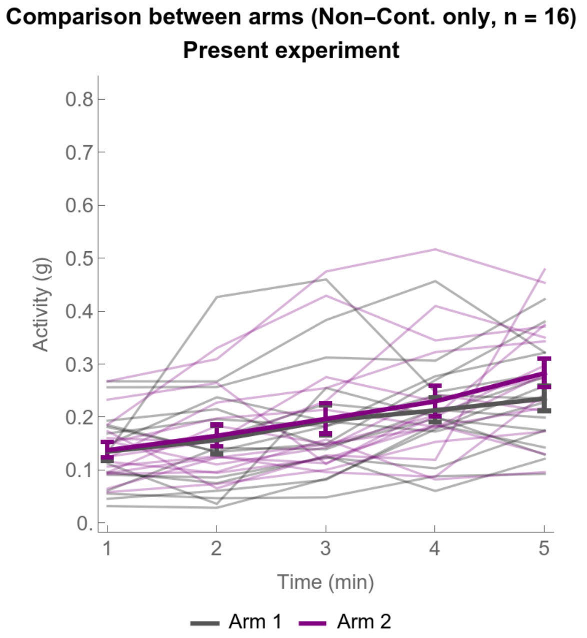

Supplementary Figure 1. *Average and individual per-minute activity. Comparison between arms (Non-Contingent condition only). The thick curves show the overall mean of the participants. The error bars are one SEM above and below the mean. Thinner curves correspond to each infant's data, on which the corresponding means are computed. The thick black curve shows mean ± SEM of the activity of Arm 1. The thick purple curve shows the same data for Arm 2. Thinner pale curves show individual data. The data of one infant with potentially far-outlying value is excluded. In the Non-Contingent condition the categories "Arm 1" and "Arm 2" were randomly attributed while approximately counterbalancing the number of left and right arms in each class.*

S.2 Verification requested by one reviewer of the ANOVAs and other analyses with unbalanced design

Prompted by a reviewer, we were asked to redo our graphs and statistics by randomly removing 4 of the 20 infants from the Contingent condition, so that there were the same number as the 16 infants in the Non-Contingent condition. There are 4845 different ways of doing this. We ran ANOVAs on all these possible subsets, comparing data of the 16 randomly-drawn participants in the Contingent with the 16 participants in the Non-Contingent condition. Each of these ANOVAs was a mixed ANOVA of the same type used in Section 3.1: with Period of the experiment (minute-1 to minute-5 means) as a within-subject factor and Condition (Contingent versus Non-Contingent) as a between-subject factor. In all of these 4845 analyses of variance, the main effects of Condition and Period (Greenhouse–Geisser corrected) were statistically significant (Condition: p-value range [0.0001 ; 0.036], η^2_G range [0.096 ; 0.31]; Period: worst observed p-value < 0.001, η^2_G range [0.07 ; 0.16]). The Condition×Period interaction (Greenhouse–Geisser corrected) had a significant effect in 2870/4845 (59%) cases. Thus, this series of analyses confirms the main effect of Condition described in Section 3.1. The fact that the Condition×Period is present only about half of the time is compatible with the idea that only the first minutes of the experiment show a strong difference.

In order to illustrate these facts we have randomly selected one of the 4845 ways of removing 4 infants, and provided the corresponding ANOVA results and redrawn the figures below.

For an easier comparison, the names of the figures in this subsection mirror the names of the corresponding figures in the Results section.

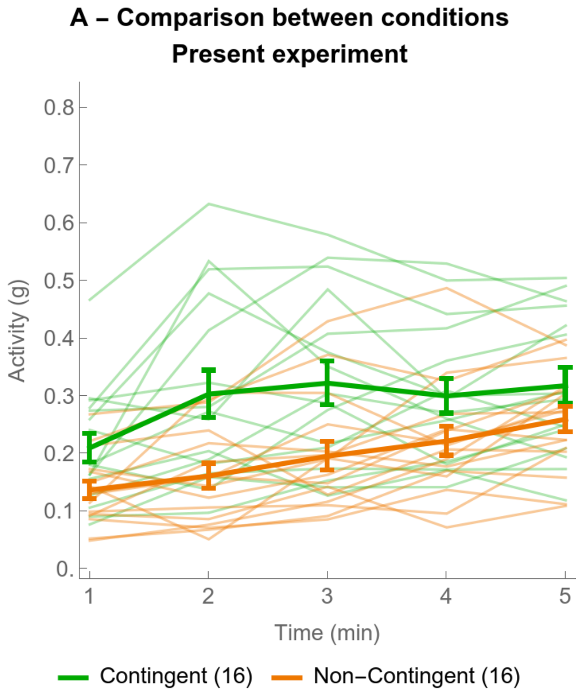

Supplementary Figure 2A. *Average and individual per-minute activity. Comparison between conditions. The thick green curve shows the mean of the pooled activity of both arms of the Contingent condition. The thick orange curve shows the same data for the Non-Contingent condition. The error bars are one SEM above and below the mean. Thinner pale curves show individual data. Present experiment: data of a random selection of 16 out of 20 infants in the Contingent condition and 16 infants in the Non-Contingent condition (one far outlier excluded).*

***Supplementary Figure* 2A** is the equivalent of **Figure 2A** of the "Results" section but with data of 16 participants in the Contingent condition; it shows, separately for the Contingent and Non-Contingent conditions, the pooled activity of both arms during the course of the experiment on a minute-by-minute basis, individually for all infants, with the means across infants shown as thicker lines. The mixed ANOVA, with Period of the experiment (minute-1 to minute-5 means) as a within-subject factor and Condition (Contingent versus Non-Contingent) as a between-subject factor, shows that the main effects of both Condition and Period are statistically significant (Condition: $F_{(1,30)} = 7.7$, $p < 0.01$, generalized eta-squared $\eta^2_G = 0.16$; Period: $F_{(2.7,79.7)} = 14.56$, with effective degrees of freedom Greenhouse–Geisser corrected, $p < 0.001$, $\eta^2_G = 0.11$). The Condition×Period interaction is here not statistically significant ($F_{(2.7,79.7)} = 2.55$, Greenhouse–Geisser corrected, $p = 0.069$, $\eta^2_G = 0.02$). However the post-hoc tests showed that, as for the analysis over 20 infants, there was a significant difference of activity between the two conditions for the second and third minute of the experiment ($t_{(30)} = 3.62$, $p = 0.027$ and $t_{(30)} = 3.11$, $p = 0.043$ Bonferroni-corrected for 5 comparisons). Overall, the replication of this analysis with 16 infants therefore shows similar conclusions.

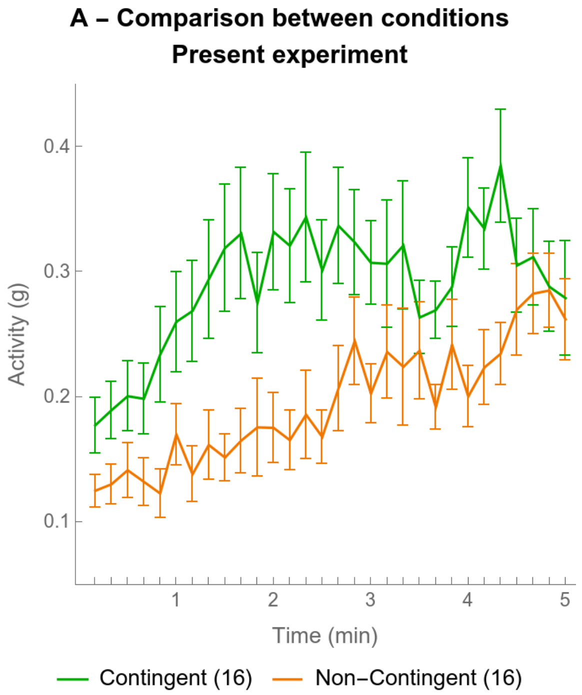

Supplementary Figure 3A. *Mean activity per 10-second bin in each condition. The mean activities were calculated for every 10-second bin over the duration of experiment. The green curve shows the means of the participants in the Contingent condition. The orange curve shows data for the participants in the Non-Contingent condition. The error bars are one SEM above and below the mean. Present experiment: data of 16 infants in the Contingent condition and 16 in the Non-Contingent condition (one far outlier excluded).*

***Supplementary Figure* 3A** is equivalent to **Figure 3A** of the “Results” section but with data of the same random subset of 16 participants as above in the Contingent condition. Exactly the same trends are seen.

***Supplementary Figure* 4** is the equivalent of **Figure 4** of the “Results” section but with data of the same random subset of 16 participants in the Contingent condition. To replicate the analysis in Section 3.3.2 for the subset of 16 infants in the Contingent condition, we re-computed the linear regressions for each individual infant for the 60 1-second bins of the first minute. The means ± SEM of the individual constants of the regressions are 0.16 ± 0.02 and 0.12 ± 0.01 for respectively the Contingent and the Non-Contingent conditions and their difference is not statistically significant ($t_{(30)} = 1.44$, $p = 0.162$ two-sided). The mean ± SEM of the individual slopes of the regressions are 0.002 ± 0.0008 and 0.0005 ± 0.0004, for respectively the Contingent and the Non-Contingent conditions, but their difference is not statistically significant ($t_{(22.02)} = 1.295$, $p = 0.104$ one-sided, Satterthwaite approximation to the 30 effective degrees of freedom was applied). There is however a difference in overall total activity (mean ± SEM) over the whole 1 minute period: the Contingent and Non-Contingent activity are respectively 0.29 ± 0.03 and 0.19 ± 0.02, and the Contingent activity is significantly greater than the Non-Contingent activity (($t_{(30)} = 2.78$, $p = 0.009$ two-sided). This re-analysis descriptively corresponds to the findings reported in Section 3.3.2; contrary to the analysis over all 20 infants the difference between slopes does not reach statistical significance for this particular subset of 16 infants, but similar to the previous analysis the total activity the Contingent condition is greater than in the Non-Contingent condition during the first minute of experiment.

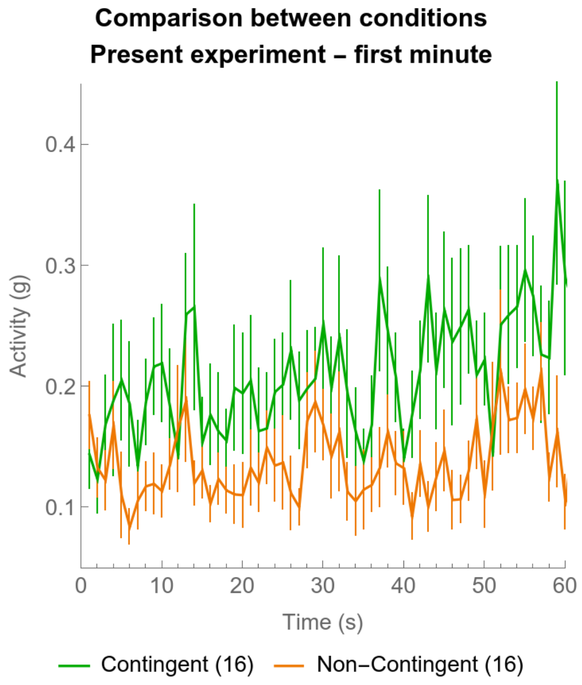

Supplementary Figure 4. *Zoom on mean activity during the first minute per 1-second bin. The mean activities were calculated for every 1-second bin over the first 60 s of the experiment. The green curve shows the means of the participants in the Contingent condition (data of 16 infants). The orange curve shows data for the Non-Contingent condition, excluding one participant with outlying values (data of 16 infants). The error bars are one SEM above and below the mean.*

S.3 10-second resolution reveals effect of age in Jacquey et al. (2020b)

The finer temporal resolution analysis of the present experiment and the experiment in Jacquey et al. (2020b) revealed aspects of the data that were hidden in the classic 1-min resolution analyses, and allowed better understanding of the phenomena at work in contingency detection. Finer temporal resolution analysis might also illuminate an aspect of Jacquey et al.'s (2020b) experiment that remained unexplained: Jacquey et al.'s (2020b) experiment was done on 4-, 6- and 8-month-old infants, and a progressively increasing sensitivity to contingency with age was expected. However, curiously neither for contingency detection, nor for arm differentiation was there a statistically significant effect of age.

Supplementary Figure 5 provides our re-analysis at 10-second temporal resolution of Jacquey et al.'s (2020b) data, for all three age groups, both for the Contingent versus Non-Contingent comparison, and for the Connected versus Unconnected arm comparison.

For the Contingent versus Non-Contingent comparison shown in the left column, at 4 months of age (**Supplementary Figure 5A1)**, there is a suggestion that infants may register the contingency at around 2 minutes into the experiment; then at 6 months of age (**Supplementary Figure 5B1**) the effect is very clear, and the curves for the Contingent versus Non-Contingent conditions clearly separate just before 2 minutes (note that these are the same curves already plotted in **Figure 2B** of the “Results” section). Curiously however at 8 months (**Supplementary Figure 5C1**), there seems again to be no difference between the curves.

For arm differentiation, the expected effect of age is on the other hand clearly present in the fine temporal resolution data. The graphs in the right column (**SupplementaryFigures 5A2,B2,C2**) show that as the infants become older, the separation between the curves for the Connected and Unconnected arms becomes progressively clearer. Wilcoxon signed-rank tests of the difference between activity scores for Connected and Unconnected arms give mean ± SEM differences at 4

months: −0.01 ± 0.01, n.s. (test statistic = 53, $p = 0.45$ two-sided); at 6 months: 0.04 ± 0.02 (test statistic = 141, $p = 0.017$ two-sided), and at 8 months: 0.06 ± 0.02 (test statistic = 132, $p = 0.009$ two-sided).

This reanalysis at finer temporal resolution of Jacquey et al.'s (2020b) data is closer to expectations than what they found at 1-min resolution. Sensitivity to contingency, as evidenced by the difference of pooled activities of both arms in the Contingent condition versus the Non-Contingent condition, does seem to develop from 4 to 6 months, although it saturates at 8 months. On the other hand, the sensitivity to contingency as evidenced by arm differentiation in the Contingent condition (that is, the difference of activity between the Connected arm and the Unconnected arm) clearly increases gradually all the way from 4 to 8 months. A possible interpretation of the saturation at 8 months of contingency detection, is that because at 8 months infants are now successfully probing the Connected arm, they do so by keeping the Unconnected arm stationary. The pooled activity of the two arms therefore does not increase in the way it did for 4- and 6-month-olds.

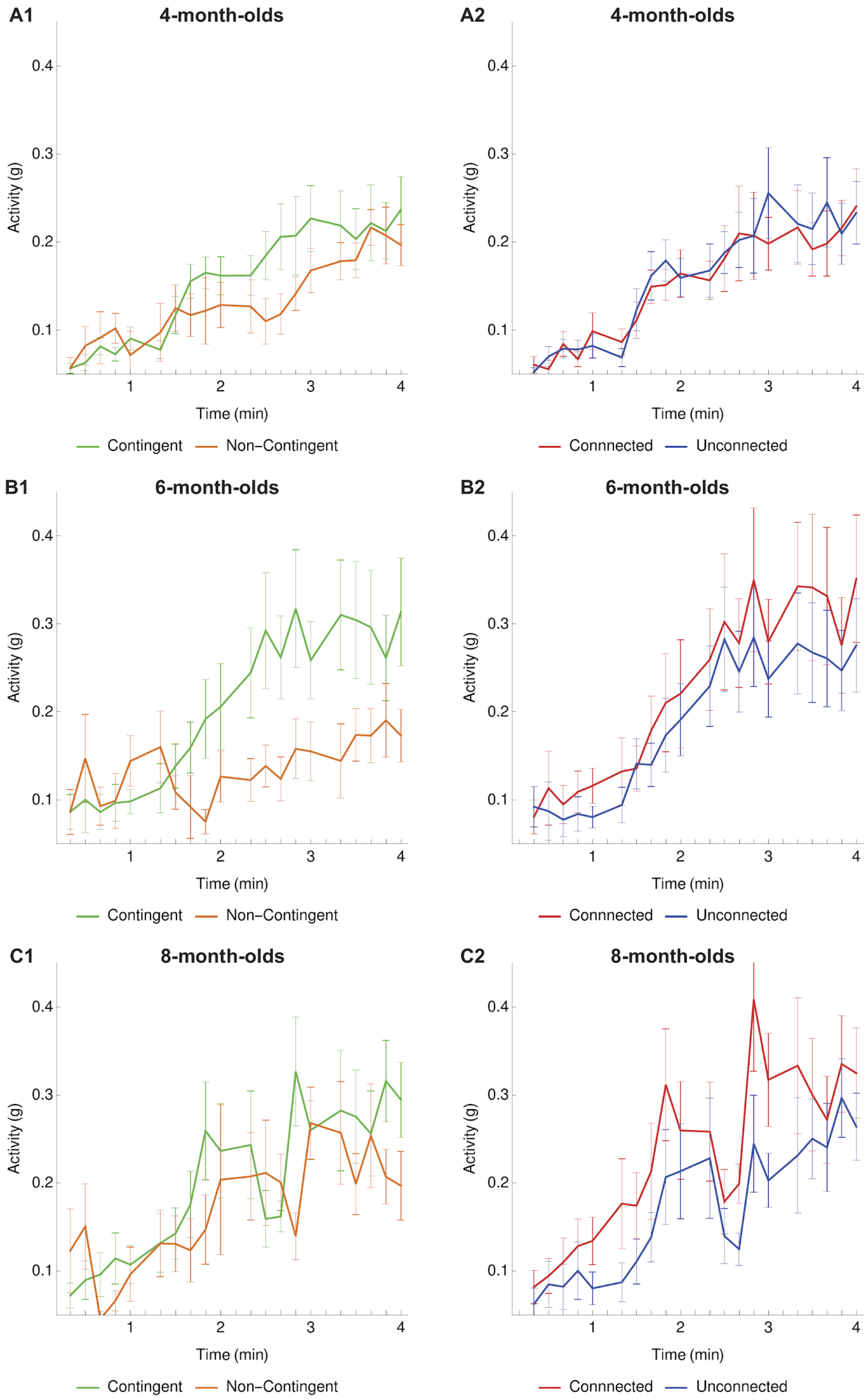

Supplementary Figure 5. *Mean activity per 10-second bin. Rows:* ***(A)*** *4-month-olds.* ***(B)*** *6-month-*

olds. ***(C)*** *8-month-olds. LEFT COLUMN: Contingent versus Non-Contingent. The mean activities pooled for both arms were calculated for every 10-second bin over the duration of the experiment excluding the bins for the attention getters. The green curve shows the means of the participants in the Contingent condition. The orange curve shows data for the Non-Contingent condition, excluding the two participants with outlying values (one 4-months-old and one 6-months-old). The error bars are one SEM above and below the mean. RIGHT COLUMN: Connected versus Unconnected. The mean activities for every 10-second bin over the duration of the experiment excluding the bins for attention getters. The red curve shows the means of the Connected arms. The blue curve shows data for the Unconnected arm. The error bars are one SEM above and below the mean.*
